# Structural Characterization and Cytotoxic Activity Evaluation of Ulvan Polysaccharides Extracted from the Green Algae *Ulva papenfussii*

**DOI:** 10.3390/md21110556

**Published:** 2023-10-25

**Authors:** Vy Ha Nguyen Tran, Maria Dalgaard Mikkelsen, Hai Bang Truong, Hieu Nhu Mai Vo, Thinh Duc Pham, Hang Thi Thuy Cao, Thuan Thi Nguyen, Anne S. Meyer, Thuy Thu Thi Thanh, Tran Thi Thanh Van

**Affiliations:** 1 NhaTrang Institute of Technology Research and Application, Vietnam Academy of Science and Technology, 02 Hung Vuong Street, Nhatrang 650000, Vietnam; havy@nitra.vast.vn (V.H.N.T.); nhuhieu@nitra.vast.vn (H.N.M.V.); ducthinh.nitra@gmail.com (T.D.P.); caohang.nitra@gmail.com (H.T.T.C.); nguyenthuan@nitra.vast.vn (T.T.N.); 2 Section for Protein Chemistry and Enzyme Technology, DTU Bioengineering-Department of Biotechnology and Biomedicine, Technical University of Denmark, 2800 Kongens Lyngby, Denmark; mdami@dtu.dk (M.D.M.); asme@dtu.dk (A.S.M.); 3 Optical Materials Research Group, Science and Technology Advanced Institute, Van Lang University, 69/68 Dang Thuy Tram Street, Ward 13, Binh Thanh District, Ho Chi Minh City 70000, Vietnam; truonghaibang@vlu.edu.vn; 4 Faculty of Applied Technology, School of Technology, Van Lang University, 69/68 Dang Thuy Tram Street, Ward 13, Binh Thanh District, Ho Chi Minh City 70000, Vietnam; 5 Institute of Chemistry, Vietnam Academy of Science and Technology, 18 Hoang Quoc Viet Street, Hanoi 10000, Vietnam; thuyttt@ich.vast.vn

**Keywords:** ulvan, *Ulva papenfussii*, structural determination, anti-tumorigenic, QSAR modeling

## Abstract

Ulvan, a sulfated heteropolysaccharide with structural and functional properties of interest for various uses, was extracted from the green seaweed *Ulva papenfussii*. *U. papenfussii* is an unexplored *Ulva* species found in the South China Sea along the central coast of Vietnam. Based on dry weight, the ulvan yield was ~15% (*w*/*w*) and the ulvan had a sulfate content of 13.4 wt%. The compositional constitution encompassed L-Rhamnose (Rha*p*), D-Xylose (Xyl*p*), D-Glucuronic acid (GlcA*p*), L-Iduronic acid (IdoA*p*), D-Galactose (Gal*p*), and D-Glucose (Glc*p*) with a molar ratio of 1:0.19:0.35:0.52:0.05:0.11, respectively. The structure of ulvan was determined using High-Performance Liquid Chromatography (HPLC), Fourier Transform Infrared Spectroscopy (FT-IR), and Nuclear Magnetic Resonance spectroscopy (NMR) methods. The results showed that the extracted ulvan comprised a mixture of two different structural forms, namely (“A3s”) with the repeating disaccharide [→4)-β-D-GlcA*p*-(1→4)-α-L-Rha*p* 3S-(1→]n, and (“B3s”) with the repeating disaccharide [→4)-α-L-IdoA*p*-(1→4)-α-L-Rha*p* 3S(1→]n. The relative abundance of A3s, and B3s was 1:1.5, respectively. The potential anticarcinogenic attributes of ulvan were evaluated against a trilogy of human cancer cell lineages. Concomitantly, Quantitative Structure–Activity Relationship (QSAR) modeling was also conducted to predict potential adverse reactions stemming from pharmacological interactions. The ulvan showed significant antitumor growth activity against hepatocellular carcinoma (IC_50_ ≈ 90 µg/mL), human breast cancer cells (IC_50_ ≈ 85 µg/mL), and cervical cancer cells (IC_50_ ≈ 67 µg/mL). The QSAR models demonstrated acceptable predictive power, and seven toxicity indications confirmed the safety of ulvan, warranting its candidacy for further in vivo testing and applications as a biologically active pharmaceutical source for human disease treatment.

## 1. Introduction

Green algae species of the genus *Ulva* represent a significant portion of the global biomass among all seaweed species [1]. *Ulva* spp., especially *Ulva lactuca*, are utilized as a food source, commonly known as sea lettuce, typically in salads and soups [2]. Carbohydrates is the dominant constituent in green algae, followed by protein, and lipids [3]. *Ulva* spp., specifically, contain notable quantities of polysaccharides (15–65%), protein (4–44%), lipids (0.3–1.6%), and ash (11–55%) in their dry biomass [2]. In particular, ulvan, a sulfated heteropolysaccharide, is found in the Ulvales order (Chlorophyta). Ulvan is primarily located within the cell walls of the algae, constituting 9 to 36% of the dry weight. Ulvan essentially consists of disaccharide repeating units of D-Glucuronic acid (GlcA*p*) or L-Iduronic acid (IdoA*p*) linked to L-Rhamnose-3-sulfate (Rha*p* 3S) and ulvanbioses of Xylose (Xyl*p*) or Xylose-2-sulfate (Xyl*p* 2S) linked to L-Rhamnose-3-sulfate (Rha*p* 3S) [4]. In this structure, sulfated Rhamnose (Rha*p*) is linked to either of the uronic acids, specifically, GlcA*p* and/or IdoA*p*, through 1,4-glycosidic bonds. These bonds are categorized as sulfate type A and type B ulvanobiouronic acid 3-sulfate, denoted as β-D-Glucuronic acid-(1,4)-α-L-Rhamnose-3-sulfate (A3s) and α-L-Iduronic acid-(1,4)-α-L-Rhamnose-3-sulfate (B3s), respectively. Disaccharide moieties comprising Xyl*p* or sulfated Xyl*p* residues leading to the formation of disaccharides called ulvanobiose 3-sulfate are referred to as U3s and U2′s3s, respectively [3,5,6]. Furthermore, GlcA*p* has been found as branches at the O-2 position of Rha*p* 3S [7,8,9]. Notably, the presence of Rha*p* and IdoA*p* appears to be distinctive to ulvan and is not found in other polysaccharides of *Ulva* sp. [2]. The composition of the monosaccharides, including Rha*p*, Xyl*p*, GlcA*p*, and IdoA*p*, interconnected through α- and β-(1,4) glycosidic bonds vary among different *Ulva* species [9,10,11,12,13]. In previous studies, we evaluated the conformational structure at the molecular level of ulvan extracted from two *Ulva* species, namely *Ulva reticulata* and *Ulva lactuca*, using Small-Angle X-ray Scattering (SAXS) methodology. The results from SAXS analysis indicated that these ulvans had a rod-like bulky chain conformation in solution [14].

Ulvan possesses a diverse array of biological activities, including anticoagulant [15,16], antioxidant [17,18], antihyperlipidemic [19,20], antimicrobial [14,21,22], antiviral [23,24], anticancer [25,26,27], and immunomodulatory properties [28,29]. Consequently, ulvan is considered a promising bioactive resource for the development of multifarious applications such as wound dressings, tissue engineering, biofilms, and drug delivery systems within pharmaceutical and alimentary sectors [30,31,32]. 

Numerous studies have investigated the potential of ulvan in inhibiting cancer cell growth, particularly breast cancer [25,33] and cervical cancer cells [25], by evaluating the effect of ulvan on toxicity and cell viability. An increasing number of research works have reported on the results of ulvan testing on human cancer cell lines, thereby delineating the potential anticancer activity of ulvan. Ulvan extracted from various species of green algae such as *U. lactuca* [25], *Ulva intestinalis* [26], *Ulva prolifera* [34], *Ulva tubulosa* [27] and *Ulva fasciata* [35] has, thus, exhibited promising anticancer properties. The antibacterial activity [14,36] as well as the anticancer activity [25,37] of ulvan extracted from *U. reticulata* and *U. lactuca* were published in our previous studies. It can be inferred that green algae species belonging to the genus *Ulva* are considered valuable sources for the isolation of ulvan with beneficial bioactivities.

To utilize ulvan as a supplement, nutraceutical, and therapeutic agent, it is imperative to elucidate the potential cytotoxicity and delineate therapeutic dosages for effective application. The assessment of toxicity typically demands the conduct of in vitro inquiries involving cellular systems as a preclinical test step before in vivo analyses utilizing controlled experimental cohorts of living organisms [29,38]. However, notably the in vivo experimentation tends to incur substantial financial costs and investments. A quantitative structure–activity relationship (QSAR) modeling approach, characterized by its mathematical foundation, has emerged as a tool to predict the toxicological and biological activities of compounds upon their chemical structures [39]. QSAR modeling can, thus, help predict and explain the structural significance of the potential effect, and in this way reduce the need for testing on animals and/or cell cultures. 

Within the *Ulva* genus, *Ulva papenfussii*, primarily distributed in the tropical coastal waters of Vietnam, was initially identified by Vietnamese researchers in 1969 (Pham Hoang Ho) [40]. Until now, none of the scientific literature has documented the extraction, composition, chemical structure, and biological activity of (ulvan) polysaccharides derived from this specific *Ulva* species. In order to examine the structural intricacies and biological functionalities of ulvan derived from *U. papenfussii*, and to provide a direction for their potential utilization as a resource in fields such as food and pharmaceuticals, our study encompasses a comprehensive analysis, based on High-Performance Liquid Chromatography—Size Exclusion Chromatography (HPLC-SEC), Fourier-Transform Infrared Spectroscopy (FT-IR), and Nuclear Magnetic Resonance spectroscopy (NMR) analyses of the fine structure of ulvan extracted from *U. papenfussii* collected from Nha Trang Bay, Vietnam. Additionally, we report the potential anticancerogenic activity of ulvan in three human cancer cell lines, including HepG2 (hepatocellular carcinoma), MCF7 (human breast cancer), and Hela (cervical cancer). Finally, we employ QSAR modeling to evaluate the toxicological attributes for safety and risk assessment.

## 2. Results

### 2.1. Chemical Composition of the Extracted Ulvan

In general, the average extraction efficiency of ulvan from *Ulva* species is 13 ± 4.3%. The extraction efficiency varies depending on the specific type of *Ulva* species. For blade species, the extraction efficiency is typically ~16% while for filamentous species, the crude extraction efficiency is ~10% [2]. We find that ulvan extracted from *U. papenfussii* (blade species) yielded an extraction efficiency of 14.8% (based on the dry weight of seaweed), which falls within the average range for blade species extraction efficiency.

The main chemical composition of ulvan extracted from *U. papenfussii* is similar to the polysaccharide extracted from other *Ulva* species with a high content of Rha*p* (45 mol% of the total carbohydrates). Additionally, it contains 23 mol% of IdoA*p*, 16 mol% of GlcA*p*, and 8.5 mol% of Xyl*p*, as well as—surprisingly—5 mol% of Glc*p*, and a negligible amount of Gal*p* (Table 1).

### 2.2. Structural Characteristics of the Extracted Ulvan from U. papenfussii

FT-IR and NMR were employed to provide detailed information about the structure of ulvan polysaccharides [41,42]. 

**Figure 1:**
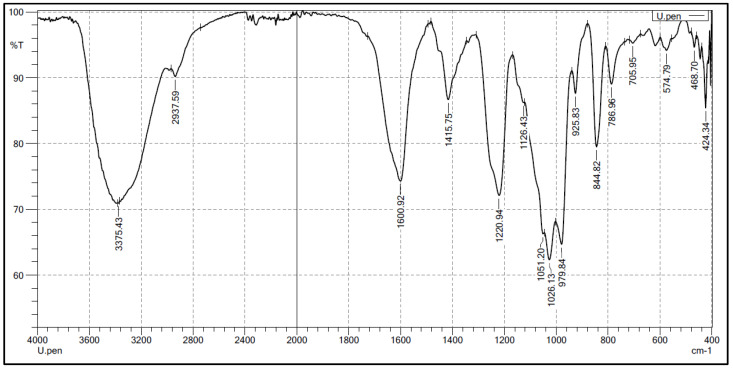
FT-IR spectrum for ulvan extracted from *U. papenfussii*. The U. pen present in FT-IR of the ulvan isolated from *U. papenfussii* and the %T is represented in the % Transmittance.

In the FT-IR spectra (Figure 1), characteristic oscillations of the sulfate ester and uronic acid groups were observed. The stretching vibrations of S-O and C-O-S bonds correspond to absorption bands at 1126 cm^−1^ and 844 cm^−1^, respectively. The signal at 786 cm^−1^ is related to the bending vibration of the C-O-S bond of the sulfate group in the equatorial position, while the signal at 1220.94 cm^−1^ corresponds to the stretching vibrations of S=O. The symmetric and asymmetric stretching vibrations of the COO- group are indicated by absorption bands at 1600 cm^−1^ and 1415 cm^−1^, respectively [41]. Additionally, the IR spectrum also exhibited absorption bands at 3375 cm^−1^, which can be assigned to the stretching vibrations of O-H in sugar molecules.

**Figure 2:**
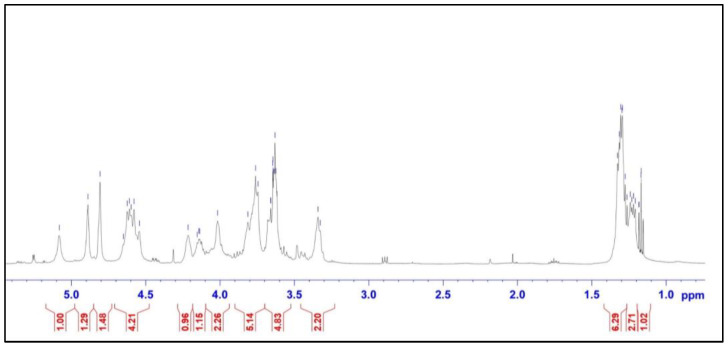
^1^H NMR spectrum of ulvan from *U. papenfussii*.

In the ^1^H NMR spectrum (Figure 2), several characteristic signals were observed in the anomeric region (5.1–4.6 ppm). Strong signals are observed at 5.08, 4.88, and 4.8 ppm, with some overlapping signals at 4.6 ppm. In the high field region, broad signals at 1.3 ppm are assigned to the methyl-C6 protons of the Rhamnopyranose moiety (C-6 methyl protons), and signals in the range of 3.32–4.21 ppm correspond to the protons of the pyranose ring.

**Figure 3:**
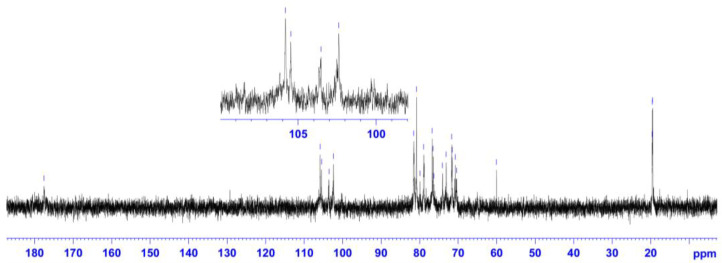
^13^C NMR spectrum of ulvan extracted from *U. papenfussii*.

The ^13^C NMR spectrum shows five strong signals for the anomeric carbons with chemical shift values (δ ppm) at 102.39, 103.53, 105.46, and 105.81, as well as signals in the range of 70.37–81.55 ppm for the pyranose ring carbons (Figure 3). In the high field region, three signals with chemical shift values at 19.50, 19.58, and 19.63 ppm are characteristic of the methyl carbons, and a signal at the low field region with a chemical shift value of 177.54 ppm is assigned to the carboxyl carbon. Additionally, the ^13^C NMR spectrum exhibits a conjoined signal at 60 ppm, characteristic of the C-5 carbon of the CH_2_ group in Xyl*p* and/or the C-6 carbon of the CH_2_ group in Glc*p* and/or Gal*p*. Thus, the ^1^H NMR and ^13^C NMR spectra confirm the presence of the ulvan components, Rha*p*, GlcA*p*, and IdoA*p* through their characteristic chemical shift values. Furthermore, the spectra also identified the presence of three chemically non-equivalent Rha*p* moieties in the ulvan sample. According to the main chemical composition analysis and 1D NMR spectrum analysis, the anomeric region should have at least six positive signals from the Xyl*p*, GlcA*p*, and IdoA*p* moieties, as well as three non-equivalent Rha*p* signals. However, in the ^1^H NMR spectrum, only five signals for the anomeric carbons and five proton signals in the anomeric region (4.6–5.1 ppm) are observed, suggesting the possibility of some overlapping signals.

**Figure 4:**
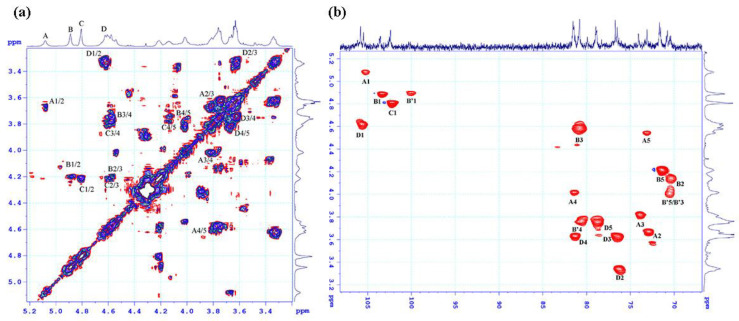
NMR spectra of ulvan extracted from *U. papenfussii* (**a**) ^1^H-^1^H COSY, A1/2 indicated the cross-peak between H-1 and H-2 of residue A, etc.; (**b**) ^1^H-^13^C HSQC, in the COSY and HSQC spectra of ulvan, cross-peaks were observed, enabling the assignment of signals to residues A, B, B’, C and D each exhibiting distinct structures as detailed in (Table 2).

To assign the spectral signals in the anomeric region (^13^C ~ 95–105 ppm/ ^1^H ~ 4.6–5.1 ppm), an HSQC analysis was performed (Figure 4). The signal at 5.08 ppm, corresponding to the signal at 105.46 ppm, was designated as A. The signal at 4.88 ppm was associated with two signals: one at 103.53 ppm and another weak signal at 100 ppm. Thus, two signals with a chemical shift of 4.88 ppm were labeled as B and B’. The signal at 4.8 ppm was assigned to the signal at 102.39 ppm, while the signals at 4.62 was connected to the signals at 105.81 ppm and were denoted as C, D, respectively. The proton signals of the moieties were determined through cross peaks in the COSY spectrum (Figure 4a) and the chemical shift values of the corresponding carbons were assigned based on the proton chemical shift values obtained from the HSQC spectrum (Figure 4b).

Based on the preceding discussion, the major signals in the NMR spectrum were assigned and presented in Table 2. The proton and carbon chemical shifts of the three moieties, B, B’, and C, confirmed their typical 6-deoxyhexopyranose form. This finding, combined with the chemical analysis results, indicates that these moieties correspond to the Rha*p* moiety. The carbon signals of C-3 and C-4 in moieties B and C displayed downfield shifts compared to the standard rhamnose spectrum, suggesting that Rha*p* may be glycosidically linked in a (1→3,4) pattern and/or sulfated at positions C-3 and C-4. Moiety B’ showed only a downfield signal for carbon C-4, indicating that it may be glycosidically linked in a (1→4) pattern and/or sulfated at this position. The chemical shift of proton H1, at 4.60 ppm, which is the signal with the lowest intensity among the proton signals in the anomer proton region, may belong to the xylose moiety. However, assigning the proton and carbon signals in the COSY and HSQC spectra is not straightforward due to the overlap of signals from both the xylose moiety and residue D. This result explains why the signals D2, D3, and D4 in the HSQC spectrum, and the strong signals D2/3, D3/4 in the HMBC spectrum of moiety D, are due to the overlap with signals from the xylose moiety. According to the chemical composition analysis, the remaining two moieties, A and D, were identified as GlcA*p* and IdoA*p*, respectively. The difference between these two acids lies in their epimeric carbon, C-5, in the pyranose ring. Based on this information and comparisons with previously published data [43,44,45], moiety A was assigned as IdoA*p*, and moiety D was assigned as GlcA*p*. Furthermore, the proton chemical shift of H1(A) at 5.08 ppm and H1(D) at 4.6 ppm confirmed that moiety A has an α-glycosidic linkage, while moiety D has a β-glycosidic linkage. The downfield shift of carbon C4 in both acids compared to the standard uronic acid spectrum confirms that both moieties may form glycosidic linkages of the (1→4) type. Therefore, moiety A was assigned as →4)-α-D-IdoA*p*-(1- and moiety D as →4)-β-D-GlcA*p*-(1-.

**Figure 5:**
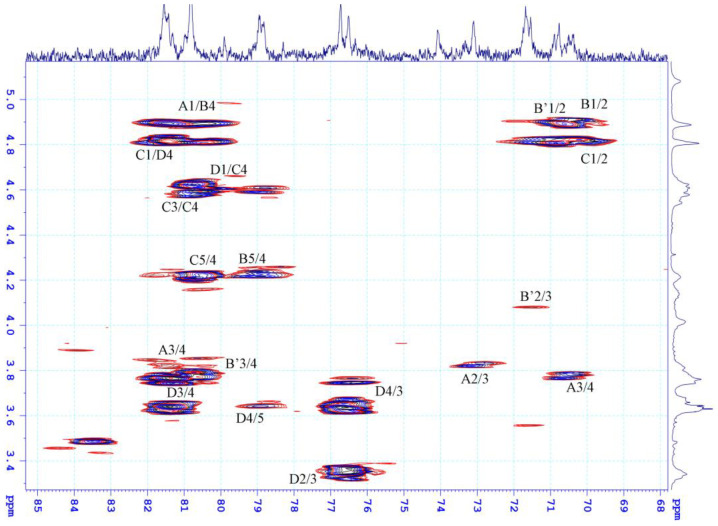
^1^H-^13^C HMBC spectrum of ulvan extracted from *U. papenfussii*. The HMBC spectra of the ulvan featured interactions, from which the signals could be assigned to A, B, B’, C and D residues with different structures (Table 2).

The determination of glycosidic linkages between sugar moieties in ulvan molecules was based on the interactions observed in the HMBC spectrum (Figure 5). The spectrum revealed interactions between carbon C1 of moiety A and proton H4 of moiety B, as well as between proton H1 of moiety B and carbon C4 of moiety A. This indicates the presence of both Rha*p*-(1→4)-α-D-IdoA*p* and α-D-IdoA*p*(1→4)-Rha*p* disaccharides in the ulvan sample. Additionally, the spectrum showed interactions between proton H1 of moiety C and carbon C4 of moiety D, and between proton H1 of moiety D and carbon C4 of moiety C, confirming the presence of Rha*p*-(1→4)-β-D-GlcA*p* and β-D-GlcA*p*-(1→4)-Rha*p* disaccharides. Thus, all moieties are connected via (1→4) glycosidic linkages. This finding explains the downfield chemical shift of the carbon C4 atoms in all moieties compared to the standard data. The increased chemical shift of proton H3 from 4.1 ppm to 4.6 ppm and carbon C3 from 71.48 ppm to ~81.0 ppm is attributed to the sulfate modification at the O-3 position, affecting neighboring bonds and direct bonding with sulfate groups, resulting in downfield-shifted correlated signals. Therefore, almost all Rha*p* moieties in the ulvan are sulfated at the O-3 position, and according to the referenced literature [45,46,47] the Rha*p* in the ulvan sample is indeed in the α-L-Rha*p* 3S form. Furthermore, the weak signal at 100 ppm assigned to the non-sulfated Rha*p* moiety indicates minimal changes in the correlated carbon-C3 signal.

### 2.3. Anticancer Activities

Cytotoxic activities of the ulvan extracted from *U. papenfussii* at various concentrations (0.8, 4, 20 and 100 µg/mL) against HepG2, MCF7 and Hela cancer cell lines were investigated (Table 3). Each data point was obtained by making three independent measurements and all data were expressed as means ± S.D (standard deviation). The IC_50_ values were estimated for HepG2, MCF7 and Hela cells to be 89.78 ± 6.55, 85.48 ± 5.75 and 66.95 ± 2.45 µg/mL, respectively, for the polysaccharide, and 0.38 ± 0.02, 0.41 ± 0.03 and 0.36 ± 0.05 µg/mL for the reference drug Ellipticine (Table 3). 

### 2.4. Toxicity Estimation Based on QSAR

The 96 h *Pimephales promelas* (fathead minnow) LC_50_, 48 h *Daphnia magna* LC_50_, 48 h *Tetrahymena pyriformis* IGC_50_, and oral rat LD_50_ were selected as acute toxicity endpoints in predicting the risk of the use of A3s and B3s ulvan. The 48 h *D. magna* LC_50_ values for A3s and B3s were calculated to be 5661 and 421 mg/L, respectively. For the *T. pyriformis* IGC_50_ endpoint, no prediction could be made, since no chemicals in the test set exceeded a minimum similarity coefficient of 0.5 for comparison purposes. The 96 h *P. promelas* LC_50_ and oral rat LD_50_ of A3s ulvan structure were computed to be 3755.9 mg/L and 2512.8 mg/kg, respectively, but were undetermined for B3s, since all the determined models showed applicability domain violation. A3s displayed a higher bioconcentration factor as compared to B3s (3.8 versus 2.44, respectively). A3s was assigned as a developmental non-toxicant, and B3s was determined to possess a negative degree of mutagenicity (computed point < 0.5). The consensus prediction for the mutagenicity of A3s and the developmental toxicity of B3s is considered unreliable, since only one prediction can be made. The typical valid model predictions and statistics are presented in the Supplementary Material.

## 3. Discussion

The composition of ulvan extracted from *U. papenfussii* was found to be similar to the polysaccharide extracted from other *Ulva* species. Notably, the [Rha*p*]:[GlcA*p* + IdoA*p* + Xyl*p*] ratio of (1:1.06) is the ideal ratio for ulvan’s structure, which suggests that the extracted ulvan is composed of ulvanobiuronic acid structures. Furthermore, the IdoA*p* content (23.4 mol%) in the extracted ulvan is significantly higher than that of ulvan extracted from other Ulva blade species (IdoA*p* ranging from 7 to 18 mol%). The sulfate content (13.4 ± 0.6%) was found to be similar in ulvan from *U. papenfussii* as compared to ulvan extracted from other Ulva blade species (13.7 ± 2.9%) [2]. The differences in ulvan extraction efficiency and monomer content can likely be explained by the species variations, harvesting region, and growth conditions of the selected seaweeds [48]. The molecular weight of ulvan extracted from *U. papenfussii* is around 5000 kDa, which is considerably higher than that of ulvan extracted from other species (10.5–312 kDa for the blade species *Ulva ohnoi* [49], 2000 kDa for the blade species *Ulva armoricana* [50] and 194 kDa for the filamentous species *U. intestinalis* [51]. The information about the molecular weight of ulvan extracted from *U. papenfussii* is very useful for guiding the selection of seaweed species and suitable technologies for the application of ulvan in the food and pharmaceutical industries [5,12,49,52].

**Figure 6:**
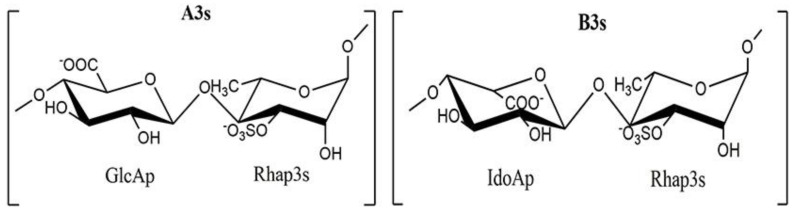
Structural characterization of ulvans extracted from *U. papenfussii*.

Based on the results of the NMR spectral analysis and chemical composition analysis, it was determined that the ulvan extracted from *U. papenfussii* is a mixture of two different structural forms, including (A3s) with the repeating disaccharide [→4)-β-D-GlcA*p*-(1→4)-α-L-Rha*p* 3S-(1→]n and (B3s) with the repeating disaccharide [→4)-α-L-IdoA*p*-(1→4)-α-L-Rha*p* 3S(1→]n (Figure 6), where the B3s form accounts for 46.5% compared to the remaining forms A3s, and the ratio of the two forms A3s:B3s is 1:1.5. The ulvan extracted from *U. papenfussii* exhibits the typical structure of ulvan polysaccharides. Similar structural mixtures have also been found in ulvan extracted from *U. ohnoi*, but with different proportions of the various structural forms, predominantly A3s and smaller amounts of B3s and U3s forms [53]. Ulvan extracted from *Ulva clathrata* seaweed is composed of the repeating disaccharide forms [→4)-β-D-GlcA*p*-(1→4)-α-L-Rha*p* 3S-(1→ and →4)-β-D-Xyl*p*-(1→4)-α-L-Rha*p* 3S-(1→, with a ratio of 4:1 between these two forms [54]. Various structural forms of ulvan often have a basic framework of A3s or B3s or U2,3s, differing in the presence of branching chains such as Xyl*p* and GlcA*p* at the C2 or C4 positions of D-glucuronosyl and L-Rhamnopyranosyl moieties, or sulfate groups that may be linked to sugar moieties and acidic moieties at the O-2 and O-3 positions. For example, ulvan extracted from *U. lactuca* primarily consists of the ulvanobiuronic acid A3s framework and a small amount of the β-GlcA*p*-(1→2)-α-Xyl*p* disaccharide [25], while the structure of ulvan extracted from *U. reticulata* primarily consists of the A3s framework [→4)-β-D-GlcA*p*-(1→4)-α-L-Rha*p* 3S-(1→], with a glucuronic acid branch at the C2 position of the β-D-Glucuronosyl moiety [14]. The F2 segmented structure with activity in ulvan extracted from *Ulva pertusa* consists of a backbone mainly composed of α-(1→4)-L-Rhamnopyranosyl, β-(1→4)-D-Glucuronosyl, β-(1→2)-L-Rhamnopyranosyl, and β-(1→4)-D-Xylopyranosyl residues with branches at the O-2 position of Rha*p*. The sulfate groups were primarily located on GlcA*p* at the O-3 position [55]. Recently, a group of authors proposed the fine structure of ulvan extracted from *U. pertusa*, which consists of 1,4-linked α-L-Rhamnopyranose, 1,3-linked α-L-Rhamnopyranose, 1,4-linked β-D-Xylopyranose, terminal β-D-Glucuronic acid (>1.5 molar), and terminal L-Iduronic acid (<0.5 molar). The terminal sugars were substituted at the C-2 and/or C-3 positions of the 1,4-linked α-L-Rhamnose residue. The sulfate groups were attached at the C-2 and C-3 positions of the Rha*p*, as well as the C-3 position of the Xyl*p* residues [56]. Therefore, the structure of ulvan is highly complex and diverse due to variations in monosaccharide composition, configuration, branching, and sulfation patterns, depending on the algal source, extraction methods, etc. This complexity likely contributes to the diverse biological properties and activities exhibited by ulvan.

The QSAR modeling calculation could not predict the toxicity of A3s and B3s for all parameters (Table 4) since some models did not reach the acceptable predictive accuracy, which was examined based on statistical external validation with three different criteria including concordance, sensitivity, and specificity. However, the validated outcomes demonstrated the non-toxicity of ulvan with A3s and B3s structures. The validated values for acute toxicity endpoints, such as 96 h *P. promelas* (fathead minnow) LC_50_, 48 h *D. magna* LC_50_, 48 h *T. pyriformis* IGC_50_, and oral rat LD_50_, were all higher than 100 mg/L, demonstrating the practically nontoxic characteristics of A3s and B3s compounds, following the acute toxicity categories as outlined by the United States Environmental Protection Agency guidelines in 2010. Ulvan with A3s and B3s structures was also predicted to be a developmental non-toxicant and negative in mutagenicity. Therefore, the QSAR-based cheminformatics approach in predicting the toxicity of ulvan in A3s and B3s structures showed promising results for safety in pharmaceutical applications, which also provided a significant reference for further in vivo testing. Previous work [25] indicated that ulvan extracted from *U. lactuca* exhibits cytotoxic activities against liver carcinoma cells (IC_50_ 29.67 ± 2.87 g/mL), human breast cancer cells (IC_50_ 25.09 ± 1.36 g/mL), and cervical cancer cells (IC_50_ 36.33 ± 3.84 g/mL). Our study provides more evidence of the putative anti-tumorigenic effects of polysaccharide from the *Ulva* genus.

## 4. Materials and Methods

### 4.1. Materials

*U. papenfussii* was collected from the Bay of Nha Trang, Khanh Hoa, Vietnam and identified by Dr. Vo Thanh Trung (Nha Trang Institute of Technology Research and Application). After collecting seaweed samples, extraneous materials such as garbage, sand, and humus were meticulously removed through rinsing with tap water. The collected specimens were then carefully dried under shaded conditions and subsequently finely ground into a powder form (Figure S1).

Standard monosaccharides including L-Rhamnose (Rha*p*), D-Galactose (Gal*p*), D-glucose (Glc*p*), D-Xylose (Xyl*p*), D-glucuronic acid (GlcA*p*), and L-iduronic acid (IdoA*p*) were purchased from Sigma-Aldrich (St. Louis, MO, USA).

### 4.2. Extraction and Purification of Ulvan

Ulvan extraction from *U. papenfussii* was conducted using the chemical method described by Bilan et al. (2002) with slight modifications [57]. The green seaweed was extracted by water at 80–90 °C for 2 h. The resulting solution was then separated by centrifugation, and the residual seaweed material underwent a second extraction using the same conditions. The combined extract was centrifuged to obtain a clear solution, and subsequently treated with Cetavlon (hexadecyltrimethylammonium bromide) to induce complete ulvan precipitation. The precipitated ulvan was then dissolved and converted into sodium salt polysaccharides. The ulvan polysaccharide was precipitated by adding 95% ethanol (EtOH) in a ulvan:EtOH ratio of 1:3 (*v*/*v*). The ulvan was solubilized in water, subjected to filtration through a 10 kDa membrane to remove salt, and finally freeze-dried [57,58,59]. The ulvan extraction procedure is illustrated in (Figure S2).

### 4.3. Chemical Analysis of Ulvan

Chemical analysis of the extracted ulvan were performed as described elsewhere [59]. For monosaccharide analysis, the ulvan samples were subjected to two-step acid hydrolysis and the resulting hydrolysates were analyzed using a Dionex ICS-5000 HPAEC-PAD (Dionex, Sunnyvale, CA, USA) system with pulsed amperometric detection (PAD) [60].

The sulfate content of the ulvan samples was determined using the turbidimetric method of Jackson and McCandless (1978) [61]. Briefly, 110 μL of hydrolysates after TFA hydrolysis were mixed with 120 μL of 8% TCA. After that, 60 μL of 2% BaCl_2_ in 15% PEG6000 reagent was added, and the mixture was allowed to stand for 35 min. The released BaSO_4_ suspension was measured at 500 nm using a microplate reader (TECAN Infinite 200, Salzburg, Austria). BaSO_4_ was used as standard to build a linear standard curve for the sulfate response.

The molecular weight (MW) of the ulvan fractions was examined using a High-Performance Size Exclusion Chromatography (HP-SEC) apparatus, composing of an Ultimate iso-3100SD pump, a WPS-3000 sampler (Dionex, Sunnyvale, CA, USA), and a RID-A refractive index detector (Shodex, Showa Denko K.K., Tokyo, Japan). A Shodex SB-806 HQ GPC column (300 × 8 mm) coupled with a Shodex SB-G guard column (50 × 6 mm) (Showa Denko K.K., Tokyo, Japan) was utilized for sample separation. Elution was carried out at a flow rate of 0.5 mL/min at 40 °C. Pullulans with molecular weight of 1, 5, 12, 110, 400, and 800 kDa were used as standards [62].

### 4.4. Structural Characterization of Ulvan

The samples (approximately 10 mg) were dissolved in 500 μL of ^2^H_2_O, and nuclear magnetic resonance (NMR) spectra were obtained using a 500 MHz Bruker Advance III HD instrument equipped with a 5 mm TCI cryoprobe and an Oxford magnet. For ^1^H NMR spectra, 6 transients were summed up, with a sampling of 16384 complex data points and a time interval of 1.7 s. ^1^H-^1^H correlation spectroscopy (COSY) was obtained by sampling 2048 × 512 complex data points for 213 ms and 53 ms in the direct and indirect dimensions, respectively. Additionally, ^1^H-^13^C heteronuclear multiple bond correlation (HMBC) spectra were acquired with a sampling of 2048 × 128 complex data points, using durations of 256 ms and 6.3 ms for the ^1^H and ^13^C dimensions, respectively. The multiplicity-edited ^1^H-^13^C heteronuclear single quantum correlation (HSQC) spectra, using adiabatic decoupling, were reported with a sampling of 2048 × 512 complex data points, using durations of 213 ms and 15.5 ms for the ^1^H and ^13^C dimensions, respectively. The ^1^H-^13^C HMBC spectra were also acquired with a sampling of 1024 × 100 complex data points, employing durations of 128 ms and 3 ms for the ^1^H and ^13^C dimensions, respectively. For the assignment spectra of the high-molecular-weight ulvan, the NMR measurements were conducted at 80 °C. All NMR spectra were processed with ample zero filling in all dimensions and baseline correction using Bruker Topspin 3.5 pl7 software. Analysis of the spectra was performed using the same software.

Fourier-transform infrared (FT-IR) spectra were acquired using a Shimadzu Afinity-1S instrument equipped with a QATR-detector, covering the wavenumber range from 400 to 4000 cm^−1^. Data were recorded in LabSolutions IR software (Version 2.27). 

### 4.5. Cytotoxic Assays

Three human cancer cell lines HepG2 (hepatocellular carcinoma), MCF7 (human breast cancer), and Hela (cervical cancer) were used for the assays (The experimental cell lines were generously provided by Prof. Dr. J. M. Pezzuto from Long Island University, USA, and Prof. Jeanette Maier from the University of Milan, Italy). The cells were cultured as a monolayer in Dulbeco’s Modified Eagle Medium (DMEM) or RPMI-1640 (depend on the cell lines) with contents including 2 mM L-glutamine, 1.5 g/L sodium bicarbonate, 4.5 g/L glucose, 10 mM HEPES, and 1.0 mM sodium pyruvate, and supplemented with Fetal Bovine Serum (FBS) 10%. The MCF7 medium was further added with 0.01 mg/mL bovine insulin. The cells were subcultured after 3–5 days with the ratio of 1:3 and incubated at 37 °C, 5% CO_2_, and 100% humidity.

Cytotoxic assays were performed according to a method developed by Monks et al. [63]. Briefly, cell lines were grown in 96-well microtiter plates with each well containing 190 μL medium. After 24 h, 10 μL of the test samples dissolved in 10% DMSO were added to the wells. The cells were then cultured for an additional 48 h, fixed with trichloroacetic acid, and stained with sulforhodamine B, followed by the determination of the optical densities at 515 nm using a Microplate Reader (BioRad, Hercules, CA, USA). The inhibitory rate of cell growth (*IR*) was calculated by the following equation:

(1) IR=100%−ODt−OD0ODc−OD0×100

where *ODt*: is the average *OD* value at day 3; *OD*_0_ is the average *OD* value at time-zero; and *ODc* is the average *OD* value of the blank DMSO control sample.

The cytotoxicity was calculated and expressed as the inhibition concentration at 50% (IC_50_ value). The IC_50_ values were determined by testing a series of sample concentrations at 100.0, 20.0, 4.0, and 0.8 μg/mL. Ellipticine was used as a positive control. Each assay was performed in triplicate, then the average value was taken, and three independent experiments were performed for the accuracy of data. The Table Curve 2Dv4 software (Version 5.01., System Software Inc., San Jose, CA, USA) was used for data analysis and for IC_50_ calculation.

### 4.6. Toxicity Prediction using QSAR Method

The OECD QSAR Toolbox (Ver. 4.4), free software developed by the Organisation for Economic Co-operation and Development, was employed to establish the QSAR models and estimate the toxicity of two major structural forms A3s and B3s of the ulvan sample extracted from *U. papenfussii.* The structural frameworks were built using Perkin Elmer Chemdraw software (Ver. 11) and imported in single mode. Seven endpoints were chosen for the toxicity evaluation, including 96-hr acute *Pimephales promelas* (fathead minnow) LC_50_, 48-h *Daphnia magna* LC_50_, 48-h *Tetrahymenas pyriformis* IGC_50_, oral rat LD_50_, bioconcentration factor, developmental toxicity, and Ames mutagenicity. More details on the selected toxicity endpoints were displayed in the Supplementary Material. An advanced hierarchical clustering approach was selected for the methodology of QSAR computation considering that other techniques, such as single model, group contribution and nearest neighbor, exhibited certain limitations including no correction for the interaction of adjacent fragments and missing estimation during external validation [64]. To ensure the robustness and predictive capability of the QSAR models, statistical external validation was undertaken [65]. The assessment procedure incorporated a comprehensive ensemble of 797 two-dimensional molecular descriptors. The attained QSAR model achieved an acceptable level of predictive efficacy solely when specific preconditions were met:

q^2^ > 0.5 (2)

R^2^ > 0.6 (3)

(R^2^ − R_o_^2^)/R^2^ < 0.1 and 0.85 ≤ k ≤ 1.15 (4)

where q^2^ is the leave-one-out correlation coefficient characterizing the training set, R^2^ signifies the correlation coefficient elucidating the relationship between the anticipated and observed toxicities within the test set, and R_o_^2^ is the correlation coefficient delineating the concordance between the projected and actual toxicities within the test set, a condition where the Y-intercept is constrained to zero.

## 5. Conclusions

Ulvan from the green seaweed *U. papenfussii* exhibits a highly diverse structure, including A3s and B3s forms, along with a notable abundance of IdoA*p* within its chemical composition (23 mol% of IdoA*p*, 16 mol% of GlcA*p*, 8.5 mol% of Xyl*p*, 5 mol% of Glc*p*, and 2.2 mol% of Gal*p*). The B3s component constitutes the majority of the ulvan structure extracted from *U. papenfussii*. This is also a distinct feature compared to the ulvan from other Ulva genus species collected along the Vietnamese coastal area, where the main backbone structure is predominantly of the A3s composition. This study has confirmed that ulvan structures vary among different Ulva genera. Based on biological activities and toxicity prediction algorithms, it has been shown that ulvan from the green seaweed *U. papenfussii* possess significant anticancer activity, while demonstrating a complete absence of cytotoxicity. This provides the essential basis for in vivo experimentation and plausible applications of this ulvan as a biologically active pharmaceutical source for human disease treatment. Additionally, ulvan extracted from *U. papenfussii* has a high molecular weight of approximately 5000 kDa and contains multiple active functional moieties, including sulfate and IdoA*p*. Together, these attributes make it a promising candidate as a bioactive compound for the management of cutaneous tissue injuries.

## Figures and Tables

**Table 1:** The monosaccharide composition of ulvans extracted from *U. papenfussii*.

Monomers		Composition (mol% of the Total Carbohydrates)
Neutral monosaccharides	Rhamnose	44.9 ± 1.5 ^1^
	Galactose	2.2 ± 0.2 ^1^
	Glucose	5.3 ± 0.4 ^1^
	Xylose	8.5 ± 0.4 ^1^
Uronic acids	Glucuronic acid	15.7 ± 1.0 ^1^
	Iduronic acid	23.4 ± 0.8 ^1^
Composition		
Sulfate (%)		13.4 ± 0.6 ^2^

^1^ The data are given in mol% (relative level) of total carbohydrates analyzed in the ulvan. ^2^ The data expressed as % of the total dry mass.

**Table 2:** Chemical shifts in ^1^H and ^13^C NMR spectra for the ulvans extracted from *U. papenfussii*.

	Residue\Atom	Chemical Shifts (ppm)					
		H1/C1	H2/C2	H3/C3	H4/C4	H5/C5	H6/C6
A	→4)-β-D-IdoA*p*-(1-	5.08	3.64	3.81	4.01	4.54	
		105.46	73.1	74.08	81.55	73.1	177.54
B	→4)-α-L-Rha*p*3S-(1-	4.88	4.15	4.57	3.74	4.21	1.3
		103.53	70.76	80.81	78.95	71.54	19.58
B’	→4)-α-L-Rha*p*-(1-	4.88	4.1	4.01	3.76	4.01	1.30
		100.00	71.54	70.76	80.81	70.76	19.5
C	→4)-α-L-Rha*p*-3S-(1-	4.80	4.21	4.59	3.76	4.21	
		102.39	71.68	79.9	80.8	70.37	19.63
D	→4)-β-D-GlcA*p*-(1-	4.62	3.34	3.64	3.65	3.74	
		105.81	76.33	76.51	81.55	78.95	177.54

**Table 3:** Cytotoxic activity of ulvans extracted from *U. papenfussii* in three different human cancer cell lines: MCF7 (human breast cancer), HepG2 (hepatocellular carcinoma), and Hela (cervical can-cer). Ellipticine was used as a positive control. Each assay was conducted in triplicate, and subsequently, the average value was computed. Three independent experiments were carried out. Data analysis and IC_50_ calculation were performed using the Table Curve 2Dv4 software.

% Cell Inhibition							
Ulvan				Ellipticine			
Conc. (µg/mL)	MCF7	HepG2	Hela	Conc. (µg/mL)	MCF7	HepG2	Hela
100	54.96 ± 2.30	52.95 ± 2.85	68.57 ± 2.43	10	107.02 ± 4.30	99.72 ± 4.70	94.18 ± 4.32
20	21.25 ± 1.32	27.21 ± 1.60	19.96 ± 1.35	2	76.76 ± 3.35	80.04 ± 3.24	88.03 ± 3.67
4	8.61 ± 0.53	24.45 ± 0.40	4.11 ± 0.30	0.4	50.24 ± 2.56	51.38 ± 2.31	47.89 ± 2.33
0.8	4.30 ± 0.03	18.11 ± 0.21	2.76 ± 0.23	0.08	23.32 ± 1.21	24.31 ± 1.33	25.67 ± 1.24
IC_50_	85.48 ± 5.75	89.78 ± 6.55	66.95 ± 2.45	IC_50_	0.41 ± 0.03	0.38 ± 0.02	0.36 ± 0.05

**Table 4:** Summary of the QSAR-calculated results for A3s and B3s structures based on seven toxicity parameters.

Toxicity Endpoints	Unit	Structural Form	
		A3s	B3s
96 h *P. promelas* LC_50_	mg/L	3755.9	N/A
48 h *D. magna* LC_50_	mg/L	5661	421
48 h *T. pyriformis* IGC_50_	mg/L	N/A	N/A
Oral rat LD_50_	mg/kg	2512.8	N/A
Bioconcentration factor		3.8	2.44
Developmental Toxicity		0.5 (non-toxicant)	N/A
Mutagenicity		N/A	0.32 (negative)

N/A: Not applicable.

## Data Availability

Not applicable.

## Supplementary Materials

The following supporting information can be downloaded at: https://www.mdpi.com/article/10.3390/md21110556/s1, Figure S1: green algae *Ulva papenfussii* collected from the Nha Trang Bay, Khanh Hoa province, Vietnam; Figure S2: extraction process of ulvan from the green seaweed *U. papenfussii*; Data S1: predicting toxicity using the QSAR method for ulvan.
