# Supplementary material for: Structural Characterization and Cytotoxic Activity Evaluation of Ulvan Polysaccharides Extracted from the Green Algae Ulva papenfussii
Source: Mar Drugs. 2023 Oct 25;21(11):556. doi: 10.3390/md21110556 (PMC10672449; doi:10.3390/md21110556)
Supplement: Supplementary file 1 [file marinedrugs-21-00556-s001.zip › marinedrugs-2648728-supplementary.pdf]

Supplementary Material

Article

Structural Characterization and Cytotoxic Activity Evaluation of Ulvan Polysaccharides extracted from Green Algae *Ulva papenfussii*

Vy Ha Nguyen Tran [1], Maria Dalgaard Mikkelsen [2], Hai Bang Truong [3,4], Hieu Nhu Mai Vo [1], Thinh Duc Pham [1], Hang Thi Thuy Cao [1], Thuan Thi Nguyen [1], Anne S. Meyer [2], Thuy Thu Thi Thanh [5] and Tran Thi Thanh Van [1, *]

[1] NhaTrang Institute of Technology Research and Application, Vietnam Academy of Science and Technology, 02 Hung Vuong Street, Nhatrang 650000, Vietnam; havy@nitra.vast.vn (V.H.N.T.); nhuhieu@nitra.vast.vn (H.N.M.V.); ducthinh.nitra@gmail.com (T.D.P.); caohang.nitra@gmail.com (H.T.T.C.); nguyenthuan@nitra.vast.vn (T.T.N.)

[2] Section for Protein Chemistry and Enzyme Technology, DTU Bioengineering-Department of Biotechnology and Biomedicine, Technical University of Denmark, 2800 Kongens Lyngby, Denmark; mdami@dtu.dk (M.D.M.); asme@dtu.dk (A.S.M.)

[3] Optical Materials Research Group, Science and Technology Advanced Institute, Van Lang University, 69/68 Dang Thuy Tram Street, Ward 13, Binh Thanh District, Ho Chi Minh City 70000, Vietnam; truonghaibang@vlu.edu.vn

[4] Faculty of Applied Technology, School of Technology, Van Lang University, 69/68 Dang Thuy Tram Street, Ward 13, Binh Thanh District, Ho Chi Minh City 70000, Vietnam

[5] Institute of Chemistry, Vietnam Academy of Science and Technology, 18 Hoang Quoc Viet Street, Hanoi 10000, Vietnam; thuyttt@ich.vast.vn

* Correspondence: vanvvlnt@yahoo.com.vn; Tel.: +84-982140850

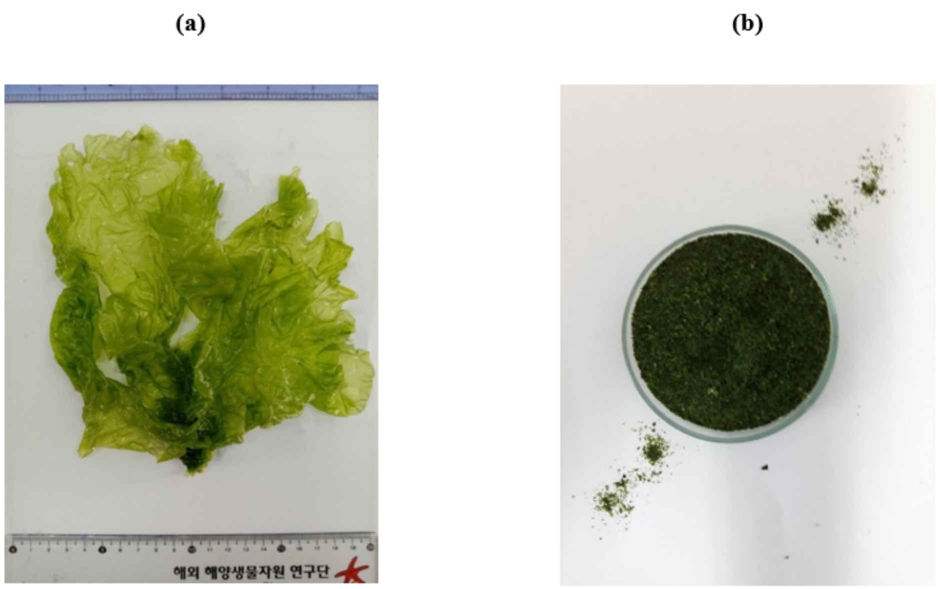

Figure S1. Green algae *Ulva papenfussii* collected from the Nha Trang Bay, Khanh Hoa province, Vietnam; (a) green algae; (b) powder of *Ulva papenfussii*.

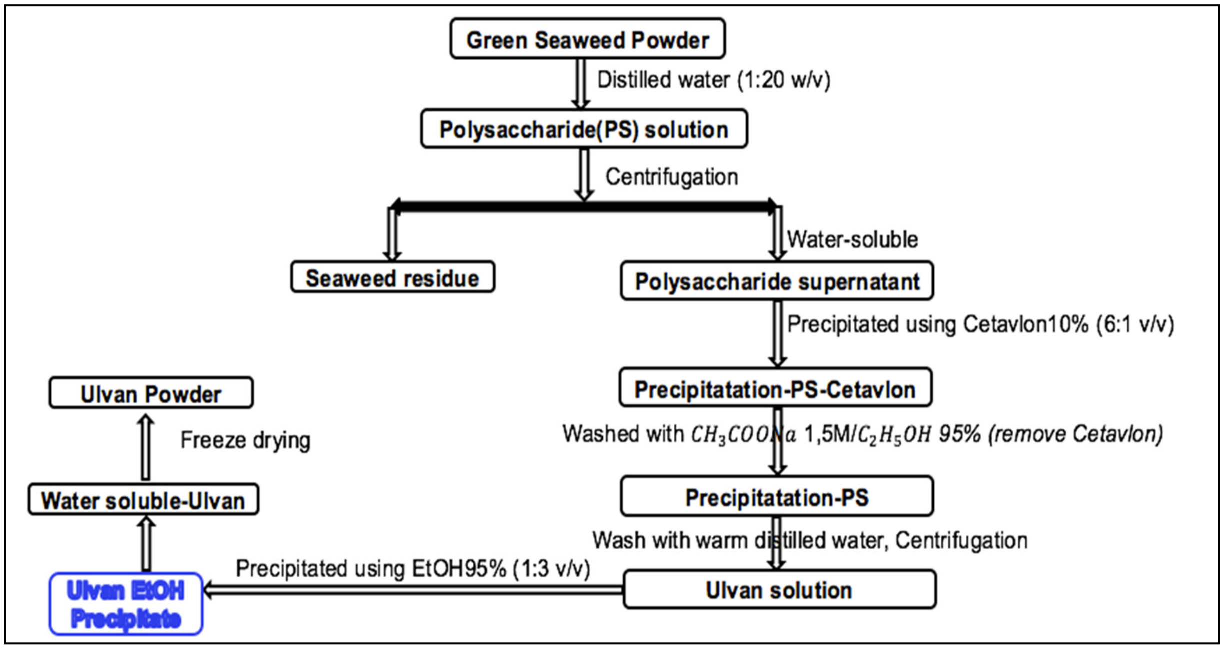

Figure S2. Extraction process of ulvan from the green seaweed *U. papenfussii*

Data S1: Predicting toxicity using the QSAR method for ulvan

1. Explanation for the toxicity endpoints used in QSAR modeling:

- 96-hr acute fathead minnow toxicity LC_{50}: concentration of the tested compound in solution (mg/L) which is lethal to half of exposed fathead minnows in 96 hr.
- 48-hr *Daphnia magna* LC_{50}: concentration of the test chemical in water in mg/L that is lethal to 50% of exposed Daphnia magna after 48 hr
- 48-hr *Tetrahymena pyriformis* IGC_{50}: concentration of the test chemical in water in mg/L that results in 50% growth inhibition to Tetrahymena pyriformis after 48 hr
- Oral rat LD_{50}: amount of chemical in mg/kg body weight that is lethal to 50% of rats after oral ingestion
- Developmental toxicity: binary indication of whether a chemical can interfere with normal development of humans or animals
- Ames mutagenicity: binary indication of whether a chemical induces revertant colony growth in any strain of *Salmonella typhimurium*.

2. Typical valid model predictions and statistics for A3s structure:

2.1. Predicted Fathead minnow LC50 (96 hr)

Prediction results

Endpoint	Experimental value	Predicted value
Fathead minnow LC_{50} (96 hr) -Log10(mol/L)	N/A	2.05
Fathead minnow LC_{50} (96 hr) mg/L	N/A	3755.86

Cluster model predictions and statistics

Cluster model	Test chemical descriptor values	Prediction interval -Log10(mol/L)	r^2	q^2	#chemicals	Applicability Domain
1310	Descriptors	6.54 ± 1.31	0.821	0.703	60	OK
1311	Descriptors	5.46 ± 1.48	0.724	0.646	92	OK
1316	Descriptors	1.76 ± 1.40	0.758	0.734	649	OK

Model # 1310

Parameter	Value
Endpoint	Fathead minnow LC_{50} (96 hr)
r^2	0.821
q^2	0.703
Number of chemicals	60
Model	1310

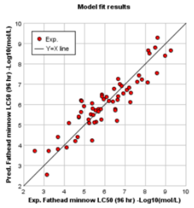

Model coefficients

Coefficient	Definition	Value	Uncertainty*
xch6	Simple 6th order chain chi index	-7.9194	2.9293
StN	Sum of (tN) E-States (StN)	0.2559	0.0811
SssS	Sum of (? S ?) E-States (SssS)	1.0083	0.6295
iedmm	Mean information content on the edge distance magnitude	1.2217	0.5758
MDEC14	Molecular distance edge between all primary and quaternary carbons	0.1283	0.1240
ATS2v	Broto-Moreau autocorrelation of a topological structure - lag 2 / weighted by atomic van der Waals volumes	3.4556	1.4594
ATS4v	Broto-Moreau autocorrelation of a topological structure - lag 4 / weighted by atomic van der Waals volumes	-2.1289	1.3255
MATS7m	Moran autocorrelation - lag 7 / weighted by atomic masses	1.4475	0.5324
MATS1e	Moran autocorrelation - lag 1 / weighted by atomic Sanderson electronegativities	3.7709	1.5065
GATS7p	Geary autocorrelation - lag 7 / weighted by atomic polarizabilities	0.7401	0.2507
XLOGP2	Wang octanol water partition coefficient squared	-0.0330	0.0130
-CH2- [aliphatic attach]	-CH2- [aliphatic attach] fragment count	-0.1286	0.0711
Model intercept	Intercept of multilinear regression model	-6.7559	2.9132

* value for 90% confidence interval

Model equation:
Fathead minnow LC50 (96 hr) = -7.9194×(xch6) + 0.2559×(StN) + 1.0083×(SssS) + 1.2217×(iedmm) + 0.1283×(MDEC14) + 3.4556×(ATS2v) - 2.1289×(ATS4v) + 1.4475×(MATS7m) + 3.7709×(MATS1e) + 0.7401×(GATS7p) - 0.0330×(XLOGP2) - 0.1286×(-CH2- [aliphatic attach]) - 6.7559

Descriptor Values

Descriptor	Value	Coefficient	Value × Coefficient
xch6	0.0907	-7.9194	-0.72
StN	0.0000	0.2559	0.00
SssS	0.0000	1.0083	0.00
iedmm	8.3545	1.2217	10.21
MDEC14	0.0000	0.1283	0.00
ATS2v	3.2849	3.4556	11.35
ATS4v	3.3025	-2.1289	-7.03
MATS7m	-0.1545	1.4475	-0.22
MATS1e	-0.1864	3.7709	-0.70
GATS7p	1.0592	0.7401	0.78
XLOGP2	11.3165	-0.0330	-0.37
-CH2- [aliphatic attach]	0.0000	-0.1286	0.00
Model intercept	1.0000	-6.7559	-6.7559
Predicted value -Log10(mol/L)			6.54

Model # 1311

Parameter	Value
Endpoint	Fathead minnow LC_{50} (96 hr)
r^2	0.724
q^2	0.646
Number of chemicals	92
Model	1311

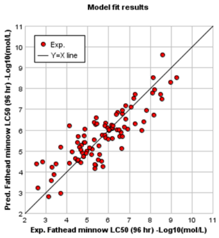

Model coefficients

Coefficient	Definition	Value	Uncertainty*
SdssC	Sum of (= C <) E-States (SdssC)	0.3796	0.1372
StN	Sum of (tN) E-States (StN)	0.2509	0.0906
SsCl	Sum of (? Cl) E-States (SsCl)	0.0207	0.0173
ib	Information bond index	0.0276	0.0208
BELm4	Lowest eigenvalue n. 4 of Burden matrix / weighted by atomic masses	-3.2211	1.2898
BELv4	Lowest eigenvalue n. 4 of Burden matrix / weighted by atomic van der Waals volumes	2.7971	1.9056
Lop	Lopping centric index	-0.5469	0.3154
ATS6m	Broto-Moreau autocorrelation of a topological structure - lag 6 / weighted by atomic masses	0.6691	0.3388
GATS6e	Geary autocorrelation - lag 6 / weighted by atomic Sanderson electronegativities	0.4558	0.3481
SRW07	Self-returning walk count of order 7	0.0010	0.0009
-S- [aliphatic attach]	-S- [aliphatic attach] fragment count	1.6066	0.7428
Model intercept	Intercept of multilinear regression model	3.3209	1.7118

* value for 90% confidence interval

Model equation:
Fathead minnow LC50 (96 hr) = 0.3796×(SdssC) + 0.2509×(StN) + 0.0207×(SsCl) + 0.0276×(ib) - 3.2211×(BELm4) + 2.7971×(BELv4) - 0.5469×(Lop) + 0.6691×(ATS6m) + 0.4558×(GATS6e) + 0.0010×(SRW07) + 1.6066×(-S- [aliphatic attach]) + 3.3209

Descriptor Values

Descriptor	Value	Coefficient	Value × Coefficient
SdssC	-1.6841	0.3796	-0.64
StN	0.0000	0.2509	0.00
SsCl	0.0000	0.0207	0.00
ib	13.7546	0.0276	0.38
BELm4	1.3396	-3.2211	-4.32
BELv4	1.4802	2.7971	4.14
Lop	1.1581	-0.5469	-0.63
ATS6m	4.1335	0.6691	2.77
GATS6e	0.9753	0.4558	0.44
SRW07	0.0000	0.0010	0.00
-S- [aliphatic attach]	0.0000	1.6066	0.00
Model intercept	1.0000	3.3209	3.3209
Predicted value -Log10(mol/L)			5.46

Model # 1316

Parameter	Value
Endpoint	Fathead minnow LC_{50} (96 hr)
r^2	0.758
q^2	0.734
Number of chemicals	649
Model	1316

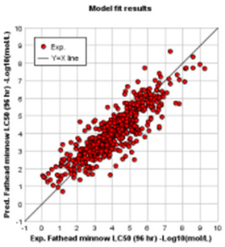

Model coefficients

Coefficient	Definition	Value	Uncertainty*
xch4	Simple 4th order chain chi index	11.6183	5.9745
ka2	Second order kappa alpha shape index	0.0685	0.0291
SsssN	Sum of (> N ?) E-States (SsssN)	-0.0866	0.0695
SdssNp	Sum of (= N+ <) E-States (SdssNp)	-0.2958	0.1253
MDEC12	Molecular distance edge between all primary and secondary carbons	-0.0624	0.0475
MDEC13	Molecular distance edge between all primary and tertiary carbons	-0.0603	0.0413
MDEC33	Molecular distance edge between all tertiary carbons	0.0967	0.0286
MDEN11	Molecular distance edge between all primary nitrogens	2.5719	1.5190
BEHm2	Highest eigenvalue n. 2 of Burden matrix / weighted by atomic masses	0.2014	0.0725
nDB	Number of double bonds	0.2845	0.0683
nR03	Number of 3-membered rings	1.0287	0.4004
ALOGP	Ghose-Crippen octanol water coefficient	0.6066	0.0507
-CH= [aromatic attach]	-CH= [aromatic attach] fragment count	0.6398	0.4642
-O- [aliphatic attach]	-O- [aliphatic attach] fragment count	-0.3795	0.1563
-OH [phosphorus attach]	-OH [phosphorus attach] fragment count	1.0172	0.3945
-CH=N	-CH=N fragment count	2.0109	0.8724
C=O(ketone, aliphatic attach)	C=O(ketone, aliphatic attach) fragment count	-0.3557	0.2257
-C(=O)- [2 nitrogen attach]	-C(=O)- [2 nitrogen attach] fragment count	-0.8516	0.4451
-COOH [aromatic attach]	-COOH [aromatic attach] fragment count	-0.9450	0.7177
-C(=O)O- [cyclic]	-C(=O)O- [cyclic] fragment count	-1.2506	0.5159
CH2=CHC(=O)O-	CH2=CHC(=O)O- fragment count	0.9931	0.5086
Model intercept	Intercept of multilinear regression model	1.4444	0.2360

* value for 90% confidence interval

Model equation:
Fathead minnow LC50 (96 hr) = 11.6183×(xch4) + 0.0685×(ka2) - 0.0866×(SsssN) - 0.2958×(SdssNp) - 0.0624×(MDEC12) - 0.0603×(MDEC13) + 0.0967×(MDEC33) + 2.5719×(MDEN11) + 0.2014×(BEHm2) + 0.2845×(nDB) + 1.0287×(nR03) + 0.6066×(ALOGP) + 0.6398×(-CH= [aromatic attach]) - 0.3795×(-O- [aliphatic attach]) + 1.0172×(-OH [phosphorus attach]) + 2.0109×(-CH=N) - 0.3557×(C=O(ketone, aliphatic attach)) - 0.8516×(-C(=O)- [2 nitrogen attach]) - 0.9450×(-COOH [aromatic attach]) - 1.2506×(-C(=O)O- [cyclic]) + 0.9931×(CH2=CHC(=O)O-) + 1.4444

Descriptor Values

Descriptor	Value	Coefficient	Value × Coefficient
xch4	0.0000	11.6183	0.00
ka2	8.2920	0.0685	0.57
SsssN	0.0000	-0.0866	0.00
SdssNp	0.0000	-0.2958	0.00
MDEC12	0.0000	-0.0624	0.00
MDEC13	2.8783	-0.0603	-0.17
MDEC33	17.6551	0.0967	1.71
MDEN11	0.0000	2.5719	0.00
BEHm2	3.8340	0.2014	0.77
nDB	3.0000	0.2845	0.85
nR03	0.0000	1.0287	0.00
ALOGP	-3.7425	0.6066	-2.27
-CH= [aromatic attach]	0.0000	0.6398	0.00
-O- [aliphatic attach]	3.0000	-0.3795	-1.14
-OH [phosphorus attach]	0.0000	1.0172	0.00
-CH=N	0.0000	2.0109	0.00
C=O(ketone, aliphatic attach)	0.0000	-0.3557	0.00
-C(=O)- [2 nitrogen attach]	0.0000	-0.8516	0.00
-COOH [aromatic attach]	0.0000	-0.9450	0.00
-C(=O)O- [cyclic]	0.0000	-1.2506	0.00
CH2=CHC(=O)O-	0.0000	0.9931	0.00
Model intercept	1.0000	1.4444	1.4444
Predicted value -Log10(mol/L)			1.76

2.2. Predicted Daphnia magna LC50 (48 hr)

Prediction results

Endpoint	Experimental value	Predicted value[b]
T. pyriformis IGC_{50} (48 hr) -Log10(mol/L)	N/A	N/A

2.3. Predicted Oral rat LD50

Prediction results

Endpoint	Experimental value	Predicted value	Prediction interval
Oral rat LD_{50} -Log10(mol/kg)	N/A	2.22	1.65 ≤ Tox ≤ 2.79
Oral rat LD_{50} mg/kg	N/A	2512.81	675.36 ≤ Tox ≤ 9349.46

Cluster model predictions and statistics

Cluster model	Test chemical descriptor values	Prediction interval -Log10(mol/kg)	r^2	q^2	#chemicals	Applicability Domain
11695	Descriptors	2.00 ± 0.79	0.796	0.712	86	OK
11750	Descriptors	1.25 ± 0.98	0.649	0.534	96	OK
11722	Descriptors	2.79 ± 0.73	0.776	0.722	93	OK
11813	Descriptors	2.41 ± 0.79	0.747	0.679	110	OK

Model # 11695

Parameter	Value
Endpoint	Oral rat LD_{50}
r^2	0.796
q^2	0.712
Number of chemicals	86
Model	11695

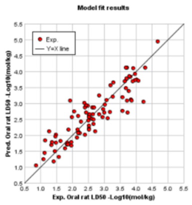

Model coefficients

Coefficient	Definition	Value	Uncertainty*
xvch5	Valence 5th order chain chi index	3.2600	2.0608
SdsCH	Sum of (= CH ?) E-States (SdsCH)	-0.0849	0.0319
icycem	Mean information on the vertex cycle matrix equality	1.1841	0.5963
MDEC22	Molecular distance edge between all secondary carbons	0.0730	0.0236
MDEC44	Molecular distance edge between all quaternary carbons	0.4774	0.4199
BEHe3	Highest eigenvalue n. 3 of Burden matrix / weighted by atomic Sanderson electronegativities	1.4600	0.9103
nR04	Number of 4-membered rings	-1.0660	0.3509
MATS7m	Moran autocorrelation - lag 7 / weighted by atomic masses	1.2755	0.4105
MATS6p	Moran autocorrelation - lag 6 / weighted by atomic polarizabilities	0.7835	0.5405
GATS6e	Geary autocorrelation - lag 6 / weighted by atomic Sanderson electronegativities	-0.5908	0.3235
C=O(ketone, aliphatic attach)	C=O(ketone, aliphatic attach) fragment count	0.3372	0.2545
-C(=O)- [olefinic attach]	-C(=O)- [olefinic attach] fragment count	0.9623	0.2448
-C(=O)O- [cyclic]	-C(=O)O- [cyclic] fragment count	0.2454	0.2428
-CF3 [aliphatic attach]	-CF3 [aliphatic attach] fragment count	0.9909	0.5804
Model intercept	Intercept of multilinear regression model	-3.4444	3.2668

* value for 90% confidence interval

Model equation:
Oral rat LD50 = 3.2600×(xvch5) - 0.0849×(SdsCH) + 1.1841×(icycem) + 0.0730×(MDEC22) + 0.4774×(MDEC44) + 1.4600×(BEHe3) - 1.0660×(nR04) + 1.2755×(MATS7m) + 0.7835×(MATS6p) - 0.5908×(GATS6e) + 0.3372×(C=O(ketone, aliphatic attach)) + 0.9623×(-C(=O)- [olefinic attach]) + 0.2454×(-C(=O)O- [cyclic]) + 0.9909×(-CF3 [aliphatic attach]) - 3.4444

Descriptor Values

Descriptor	Value	Coefficient	Value × Coefficient
xvch5	0.0000	3.2600	0.00
SdsCH	0.0000	-0.0849	0.00
icycem	0.7642	1.1841	0.90
MDEC22	0.0000	0.0730	0.00
MDEC44	0.0000	0.4774	0.00
BEHe3	3.6388	1.4600	5.31
nR04	0.0000	-1.0660	0.00
MATS7m	-0.1545	1.2755	-0.20
MATS6p	-0.0005	0.7835	-0.00
GATS6e	0.9753	-0.5908	-0.58
C=O(ketone, aliphatic attach)	0.0000	0.3372	0.00
-C(=O)- [olefinic attach]	0.0000	0.9623	0.00
-C(=O)O- [cyclic]	0.0000	0.2454	0.00
-CF3 [aliphatic attach]	0.0000	0.9909	0.00
Model intercept	1.0000	-3.4444	-3.4444
Predicted value -Log10(mol/kg)			2.00

Model # 11750

Parameter	Value
Endpoint	Oral rat LD_{50}
r^2	0.649
q^2	0.534
Number of chemicals	96
Model	11750

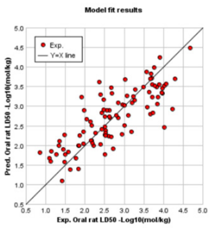

Model coefficients

Coefficient	Definition	Value	Uncertainty*
xch3	Simple 3rd order chain chi index	1.4717	1.1391
xvch5	Valence 5th order chain chi index	7.6974	2.3423
MDEC23	Molecular distance edge between all secondary and tertiary carbons	0.0546	0.0177
MDEC44	Molecular distance edge between all quaternary carbons	0.6888	0.4597
MATS7m	Moran autocorrelation - lag 7 / weighted by atomic masses	0.9273	0.4300
MATS6v	Moran autocorrelation - lag 6 / weighted by atomic van der Waals volumes	0.9081	0.5911
MATS4p	Moran autocorrelation - lag 4 / weighted by atomic polarizabilities	0.9733	0.7571
-O- [2 aromatic attach]	-O- [2 aromatic attach] fragment count	0.6503	0.3494
-C(=O)- [nitrogen, aliphatic attach]	-C(=O)- [nitrogen, aliphatic attach] fragment count	-0.3292	0.2031
-C(=O)O- [nitrogen attach]	-C(=O)O- [nitrogen attach] fragment count	0.9255	0.5557
-CF3 [aliphatic attach]	-CF3 [aliphatic attach] fragment count	0.8998	0.7123
Model intercept	Intercept of multilinear regression model	1.5446	0.3002

* value for 90% confidence interval

Model equation:
Oral rat LD50 = 1.4717×(xch3) + 7.6974×(xvch5) + 0.0546×(MDEC23) + 0.6888×(MDEC44) + 0.9273×(MATS7m) + 0.9081×(MATS6v) + 0.9733×(MATS4p) + 0.6503×(-O- [2 aromatic attach]) - 0.3292×(-C(=O)- [nitrogen, aliphatic attach]) + 0.9255×(-C(=O)O- [nitrogen attach]) + 0.8998×(-CF3 [aliphatic attach]) + 1.5446

Descriptor Values

Descriptor	Value	Coefficient	Value × Coefficient
xch3	0.0000	1.4717	0.00
xvch5	0.0000	7.6974	0.00
MDEC23	0.0000	0.0546	0.00
MDEC44	0.0000	0.6888	0.00
MATS7m	-0.1545	0.9273	-0.14
MATS6v	-0.0002	0.9081	-0.00
MATS4p	-0.1539	0.9733	-0.15
-O- [2 aromatic attach]	0.0000	0.6503	0.00
-C(=O)- [nitrogen, aliphatic attach]	0.0000	-0.3292	0.00
-C(=O)O- [nitrogen attach]	0.0000	0.9255	0.00
-CF3 [aliphatic attach]	0.0000	0.8998	0.00
Model intercept	1.0000	1.5446	1.5446
Predicted value -Log10(mol/kg)			1.25

2.4. Bioconcentration factor

Prediction results

Endpoint	Experimental value	Predicted value	Prediction interval
Bioconcentration factor Log10	N/A	0.58	-0.67 ≤ Tox ≤ 1.84
Bioconcentration factor	N/A	3.84	0.22 ≤ Tox ≤ 68.41

Cluster model predictions and statistics

Cluster model	Test chemical descriptor values	Prediction interval Log10	r^2	q^2	#chemicals	Applicability Domain
1079	Descriptors	0.58 ± 1.25	0.748	0.715	343	OK

Model # 1079

Parameter	Value
Endpoint	Bioconcentration factor
r^2	0.748
q^2	0.715
Number of chemicals	343
Model	1079

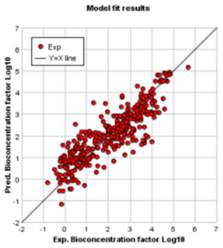

Model coefficients

Coefficient	Definition	Value	Uncertainty*
xvp10	Valence 10th order path chi index	-0.9711	0.3808
SdO_acnt	Count of (= O) (SdO_acnt)	-0.3265	0.1339
SHsNH2	Sum of ([? NH2]) hydrogen E-States (SHsNH2)	-0.1621	0.1023
icycmm	Mean information on the vertex cycle matrix magnitude	0.1450	0.0607
MDEC13	Molecular distance edge between all primary and tertiary carbons	0.0849	0.0301
MDEN22	Molecular distance edge between all secondary nitrogens	-0.2066	0.1099
BEHp2	Highest eigenvalue n. 2 of Burden matrix / weighted by atomic polarizabilities	1.4370	0.3704
MAXDN	Maximal electrotopological negative variation	0.1665	0.0529
nDB	Number of double bonds	-0.2134	0.0990
MATS1v	Moran autocorrelation - lag 1 / weighted by atomic van der Waals volumes	1.1491	0.2153
GATS5v	Geary autocorrelation - lag 5 / weighted by atomic van der Waals volumes	-0.3254	0.1647
GATS3p	Geary autocorrelation - lag 3 / weighted by atomic polarizabilities	0.5738	0.1874
Ui	unsaturation index	-0.3154	0.0875
-Cl [aromatic attach]	-Cl [aromatic attach] fragment count	0.3098	0.0567
-C(=O)- [2 aromatic attach]	-C(=O)- [2 aromatic attach] fragment count	0.4964	0.2100
-COOH [aliphatic attach]	-COOH [aliphatic attach] fragment count	-0.6537	0.3452
Model intercept	Intercept of multilinear regression model	-3.1893	1.1958

* value for 90% confidence interval

Model equation:
Bioconcentration factor = -0.9711×(xvp10) - 0.3265×(SdO_acnt) - 0.1621×(SHsNH2) + 0.1450×(icycmm) + 0.0849×(MDEC13) - 0.2066×(MDEN22) + 1.4370×(BEHp2) + 0.1665×(MAXDN) - 0.2134×(nDB) + 1.1491×(MATS1v) - 0.3254×(GATS5v) + 0.5738×(GATS3p) - 0.3154×(Ui) + 0.3098×(-Cl [aromatic attach]) + 0.4964×(-C(=O)- [2 aromatic attach]) - 0.6537×(-COOH [aliphatic attach]) - 3.1893

Descriptor Values

Descriptor	Value	Coefficient	Value × Coefficient
xvp10	0.0958	-0.9711	-0.09
SdO_acnt	3.0000	-0.3265	-0.98
SHsNH2	0.0000	-0.1621	0.00
icycmm	3.5850	0.1450	0.52
MDEC13	2.8783	0.0849	0.24
MDEN22	0.0000	-0.2066	0.00
BEHp2	3.6289	1.4370	5.21
MAXDN	6.0447	0.1665	1.01
nDB	3.0000	-0.2134	-0.64
MATS1v	-0.3091	1.1491	-0.36
GATS5v	0.8656	-0.3254	-0.28
GATS3p	0.7358	0.5738	0.42
Ui	2.0000	-0.3154	-0.63
-Cl [aromatic attach]	0.0000	0.3098	0.00
-C(=O)- [2 aromatic attach]	0.0000	0.4964	0.00
-COOH [aliphatic attach]	1.0000	-0.6537	-0.65
Model intercept	1.0000	-3.1893	-3.1893
Predicted value Log10			0.58

2.5. Predicted Developmental Toxicity

Prediction results

Endpoint	Experimental value	Predicted value
Developmental Toxicity value	N/A	0.50
Developmental Toxicity result	N/A	Developmental NON-toxicant

Cluster model predictions and statistics

Cluster model	Test chemical descriptor values	Predicted value	Concordance	Sensitivity	Specificity	#chemicals	Applicability Domain
447	Descriptors	0.32	1.000	1.000	1.000	34	OK
450	Descriptors	0.88	0.948	1.000	0.885	58	OK
451	Descriptors	0.59	0.910	0.977	0.745	188	OK
452	Descriptors	0.30	0.841	0.936	0.629	227	OK

Model # 447

Parameter	Value
Endpoint	Developmental Toxicity
Concordance	1.000
Sensitivity	1.000
Specificity	1.000
Number of chemicals	34
Model	447

Model coefficients

Coefficient	Definition	Value	Uncertainty*
MDEC33	Molecular distance edge between all tertiary carbons	0.0249	0.0069
MATS5m	Moran autocorrelation - lag 5 / weighted by atomic masses	1.3442	0.4887
MATS6m	Moran autocorrelation - lag 6 / weighted by atomic masses	0.4615	0.3631
MATS5p	Moran autocorrelation - lag 5 / weighted by atomic polarizabilities	0.6944	0.2663
GATS3v	Geary autocorrelation - lag 3 / weighted by atomic van der Waals volumes	0.8598	0.4367
-NH- [aromatic attach]	-NH- [aromatic attach] fragment count	0.6840	0.2544
Model intercept	Intercept of multilinear regression model	-0.8745	0.3107

* value for 90% confidence interval

Model equation:
Developmental Toxicity = 0.0249×(MDEC33) + 1.3442×(MATS5m) + 0.4615×(MATS6m) + 0.6944×(MATS5p) + 0.8598×(GATS3v) + 0.6840×(-NH- [aromatic attach]) - 0.8745

Descriptor Values

Descriptor	Value	Coefficient	Value × Coefficient
MDEC33	17.6551	0.0249	0.44
MATS5m	-0.0370	1.3442	-0.05
MATS6m	-0.0125	0.4615	-0.01
MATS5p	0.0794	0.6944	0.06
GATS3v	0.8770	0.8598	0.75
-NH- [aromatic attach]	0.0000	0.6840	0.00
Model intercept	1.0000	-0.8745	-0.8745
Predicted value			0.32

Model # 451

Parameter	Value
Endpoint	Developmental Toxicity
Concordance	0.910
Sensitivity	0.977
Specificity	0.745
Number of chemicals	188
Model	451

Model coefficients

Coefficient	Definition	Value	Uncertainty*
xvch7	Valence 7th order chain chi index	5.9616	4.8767
SHsOH	Sum of ([? OH]) hydrogen E-States (SHsOH)	-0.0302	0.0165
Hmax	Maximum hydrogen E-State value in molecule.	0.1807	0.1141
ic	Information content	-0.0096	0.0067
icycem	Mean information on the vertex cycle matrix equality	0.4907	0.2325
MDEN33	Molecular distance edge between all tertiary nitrogens	0.4846	0.2882
MATS2p	Moran autocorrelation - lag 2 / weighted by atomic polarizabilities	0.4541	0.2440
GATS1v	Geary autocorrelation - lag 1 / weighted by atomic van der Waals volumes	-0.3367	0.2083
GATS4p	Geary autocorrelation - lag 4 / weighted by atomic polarizabilities	-0.2506	0.1330
-CH3 [aromatic attach]	-CH3 [aromatic attach] fragment count	-0.1317	0.0915
-CH= [aromatic attach]	-CH= [aromatic attach] fragment count	-0.4264	0.2350
>C= [aromatic attach]	>C= [aromatic attach] fragment count	0.1666	0.1110
AC	AC fragment count	0.0304	0.0209
-NH2 [aliphatic attach]	-NH2 [aliphatic attach] fragment count	0.1766	0.0731
-S- [aliphatic attach]	-S- [aliphatic attach] fragment count	-0.8136	0.1543
-S(=O)(=O)- [aliphatic attach]	-S(=O)(=O)- [aliphatic attach] fragment count	0.5328	0.2531
Model intercept	Intercept of multilinear regression model	0.4765	0.4643

* value for 90% confidence interval

Model equation:
Developmental Toxicity = 5.9616×(xvch7) - 0.0302×(SHsOH) + 0.1807×(Hmax) - 0.0096×(ic) + 0.4907×(icycem) + 0.4846×(MDEN33) + 0.4541×(MATS2p) - 0.3367×(GATS1v) - 0.2506×(GATS4p) - 0.1317×(-CH3 [aromatic attach]) - 0.4264×(-CH= [aromatic attach]) + 0.1666×(>C= [aromatic attach]) + 0.0304×(AC) + 0.1766×(-NH2 [aliphatic attach]) - 0.8136×(-S- [aliphatic attach]) + 0.5328×(-S(=O)(=O)- [aliphatic attach]) + 0.4765

Descriptor Values

Descriptor	Value	Coefficient	Value × Coefficient
xvch7	0.0000	5.9616	0.00
SHsOH	19.9085	-0.0302	-0.60
Hmax	3.0165	0.1807	0.55
ic	2.0000	-0.0096	-0.02
icycem	0.7642	0.4907	0.38
MDEN33	0.0000	0.4846	0.00
MATS2p	0.0178	0.4541	0.01
GATS1v	1.3059	-0.3367	-0.44
GATS4p	1.1577	-0.2506	-0.29
-CH3 [aromatic attach]	0.0000	-0.1317	0.00
-CH= [aromatic attach]	0.0000	-0.4264	0.00
>C= [aromatic attach]	0.0000	0.1666	0.00
AC	0.0000	0.0304	0.00
-NH2 [aliphatic attach]	0.0000	0.1766	0.00
-S- [aliphatic attach]	0.0000	-0.8136	0.00
-S(=O)(=O)- [aliphatic attach]	1.0000	0.5328	0.53
Model intercept	1.0000	0.4765	0.4765
Predicted value			0.59

Model # 452

Parameter	Value
Endpoint	Developmental Toxicity
Concordance	0.841
Sensitivity	0.936
Specificity	0.629
Number of chemicals	227
Model	452

Model coefficients

Coefficient	Definition	Value	Uncertainty*
xvch9	Valence 9th order chain chi index	14.8609	12.2997
SsaaN	Sum of (saaN) E-States (SsaaN)	-0.1686	0.1540
SHsOH	Sum of ([? OH]) hydrogen E-States (SHsOH)	-0.0365	0.0165
Hmax	Maximum hydrogen E-State value in molecule.	0.2196	0.0975
ib	Information bond index	0.0101	0.0043
MDEN11	Molecular distance edge between all primary nitrogens	0.6064	0.2997
MDEN33	Molecular distance edge between all tertiary nitrogens	0.4425	0.2566
BEHm1	Highest eigenvalue n. 1 of Burden matrix / weighted by atomic masses	0.0300	0.0296
ARR	Aromatic ratio	-0.3353	0.2102
MATS3v	Moran autocorrelation - lag 3 / weighted by atomic van der Waals volumes	-0.2669	0.1766
GATS2p	Geary autocorrelation - lag 2 / weighted by atomic polarizabilities	-0.3290	0.2014
GATS5p	Geary autocorrelation - lag 5 / weighted by atomic polarizabilities	-0.1879	0.0981
-S- [aliphatic attach]	-S- [aliphatic attach] fragment count	-0.8749	0.1549
Model intercept	Intercept of multilinear regression model	0.5360	0.2801

* value for 90% confidence interval

Model equation:
Developmental Toxicity = 14.8609×(xvch9) - 0.1686×(SsaaN) - 0.0365×(SHsOH) + 0.2196×(Hmax) + 0.0101×(ib) + 0.6064×(MDEN11) + 0.4425×(MDEN33) + 0.0300×(BEHm1) - 0.3353×(ARR) - 0.2669×(MATS3v) - 0.3290×(GATS2p) - 0.1879×(GATS5p) - 0.8749×(-S- [aliphatic attach]) + 0.5360

Descriptor Values

Descriptor	Value	Coefficient	Value × Coefficient
xvch9	0.0000	14.8609	0.00
SsaaN	0.0000	-0.1686	0.00
SHsOH	19.9085	-0.0365	-0.73
Hmax	3.0165	0.2196	0.66
ib	13.7546	0.0101	0.14
MDEN11	0.0000	0.6064	0.00
MDEN33	0.0000	0.4425	0.00
BEHm1	4.5841	0.0300	0.14
ARR	0.0000	-0.3353	0.00
MATS3v	0.0787	-0.2669	-0.02
GATS2p	0.7586	-0.3290	-0.25
GATS5p	0.9410	-0.1879	-0.18
-S- [aliphatic attach]	0.0000	-0.8749	0.00
Model intercept	1.0000	0.5360	0.5360
Predicted value			0.30

2.6. Predicted Mutagenicity

Prediction results

Endpoint	Experimental value	Predicted value[b]
Mutagenicity value	N/A	N/A
Mutagenicity result	N/A	N/A

[b]A prediction cannot be made

No statistically valid models were selected by the hierarchical clustering algorithm for this compound

Cluster models with applicability domain violation

Cluster model	Test chemical descriptor values	Predicted value	Concordance	Sensitivity	Specificity	#chemicals	Applicability Domain
8727	Descriptors	-0.08	1.000	1.000	1.000	5	Rmax constraint not met
8492	Descriptors	1.76	0.947	0.857	1.000	19	Rmax constraint not met
8928	Descriptors	0.08	0.972	1.000	0.933	36	Rmax constraint not met
9071	Descriptors	0.48	0.965	0.833	1.000	86	Model ellipsoid constraint not met

Descriptor values for test chemical

3. Typical valid model predictions and statistics for B3s structure:

3.1. Predicted Fathead minnow LC50 (96 hr)

Prediction results

Endpoint	Experimental value	Predicted value[b]
Fathead minnow LC_{50} (96 hr) -Log10(mol/L)	N/A	N/A
Fathead minnow LC_{50} (96 hr) mg/L	N/A	N/A

[b]No prediction could be made

Individual Predictions

Method	Predicted value -Log10(mol/L)
Hierarchical clustering	N/A
Single model	N/A
Group contribution	N/A
Nearest neighbor	N/A

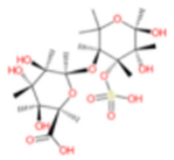

3.2. Predicted Daphnia magna LC50 (48 hr)

Prediction results

Endpoint	Experimental value	Predicted value
Daphnia magna LC_{50} (48 hr) -Log10(mol/L)	N/A	3.12
Daphnia magna LC_{50} (48 hr) mg/L	N/A	421.04

Cluster model predictions and statistics

Cluster model	Test chemical descriptor values	Prediction interval -Log10(mol/L)	r^2	q^2	#chemicals	Applicability Domain
854	Descriptors	4.73 ± 1.30	0.725	0.571	7	OK
862	Descriptors	1.69 ± 1.60	0.707	0.656	432	OK

Model # 854

Parameter	Value
Endpoint	Daphnia magna LC_{50} (48 hr)
r^2	0.725
q^2	0.571
Number of chemicals	7
Model	854

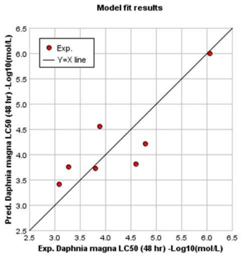

Model coefficients

Coefficient	Definition	Value	Uncertainty*
MWC09	Molecular walk count of order 9	2.1019	1.1676
Model intercept	Intercept of multilinear regression model	-21.2846	14.1706

* value for 90% confidence interval

Model equation:
Daphnia magna LC50 (48 hr) = 2.1019×(MWC09) - 21.2846

Descriptor Values

Descriptor	Value	Coefficient	Value × Coefficient
MWC09	12.3758	2.1019	26.01
Model intercept	1.0000	-21.2846	-21.2846
Predicted value -Log10(mol/L)			4.73

Model # 862

Parameter	Value
Endpoint	Daphnia magna LC_{50} (48 hr)
r^2	0.707
q^2	0.656
Number of chemicals	432
Model	862

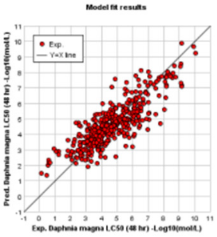

Model coefficients

Coefficient	Definition	Value	Uncertainty*
SssCH2	Sum of (? CH2 ?) E-States (SssCH2)	-0.1098	0.0302
SddC	Sum of (= C =) E-States (SddC)	0.5315	0.2260
StN	Sum of (tN) E-States (StN)	0.1020	0.0418
SsNH2_acnt	Count of (? NH2) (SsNH2_acnt)	0.7574	0.2171
SdsssP_acnt	Count of (dsssP) (SdsssP_acnt)	1.0683	0.6658
SaaS_acnt	Count of (aaS) (SaaS_acnt)	-0.9442	0.4854
Qsv	Average of Qs and Qv	0.6629	0.3714
MDEN12	Molecular distance edge between all primary and secondary nitrogens	-0.3705	0.3269
BEHm1	Highest eigenvalue n. 1 of Burden matrix / weighted by atomic masses	0.3299	0.1476
TIE	E-state topological parameter	-0.0008	0.0008
ATS5p	Broto-Moreau autocorrelation of a topological structure - lag 5 / weighted by atomic polarizabilities	0.1089	0.0820
MATS6m	Moran autocorrelation - lag 6 / weighted by atomic masses	-0.2993	0.2428
MATS4v	Moran autocorrelation - lag 4 / weighted by atomic van der Waals volumes	-0.2083	0.1938
MATS6v	Moran autocorrelation - lag 6 / weighted by atomic van der Waals volumes	0.5849	0.2437
MATS8v	Moran autocorrelation - lag 8 / weighted by atomic van der Waals volumes	0.4000	0.2629
GATS1p	Geary autocorrelation - lag 1 / weighted by atomic polarizabilities	-0.3492	0.2344
GATS3p	Geary autocorrelation - lag 3 / weighted by atomic polarizabilities	0.3034	0.1682
SRW07	Self-returning walk count of order 7	0.0014	0.0006
ALOGP	Ghose-Crippen octanol water coefficient	0.5107	0.0592
=C [aliphatic attach]	=C [aliphatic attach] fragment count	0.3600	0.1521
-O- [phosphorus attach]	-O- [phosphorus attach] fragment count	0.8688	0.3389
-OH [aromatic attach]	-OH [aromatic attach] fragment count	0.2668	0.2267
-CH=N	-CH=N fragment count	1.0449	0.6211
-C(=O)O- [aliphatic attach]	-C(=O)O- [aliphatic attach] fragment count	0.4000	0.2854
-C(=O)O- [nitrogen attach]	-C(=O)O- [nitrogen attach] fragment count	0.9850	0.4133
-CCl3 [aliphatic attach]	-CCl3 [aliphatic attach] fragment count	0.7305	0.5572
Model intercept	Intercept of multilinear regression model	0.9039	0.6882

* value for 90% confidence interval

Model equation:
Daphnia magna LC50 (48 hr) = -0.1098×(SssCH2) + 0.5315×(SddC) + 0.1020×(StN) + 0.7574×(SsNH2_acnt) + 1.0683×(SdsssP_acnt) - 0.9442×(SaaS_acnt) + 0.6629×(Qsv) - 0.3705×(MDEN12) + 0.3299×(BEHm1) - 0.0008×(TIE) + 0.1089×(ATS5p) - 0.2993×(MATS6m) - 0.2083×(MATS4v) + 0.5849×(MATS6v) + 0.4000×(MATS8v) - 0.3492×(GATS1p) + 0.3034×(GATS3p) + 0.0014×(SRW07) + 0.5107×(ALOGP) + 0.3600×(=C [aliphatic attach]) + 0.8688×(-O- [phosphorus attach]) + 0.2668×(-OH [aromatic attach]) + 1.0449×(-CH=N) + 0.4000×(-C(=O)O- [aliphatic attach]) + 0.9850×(-C(=O)O- [nitrogen attach]) + 0.7305×(-CCl3 [aliphatic attach]) + 0.9039

Descriptor Values

Descriptor	Value	Coefficient	Value × Coefficient
SssCH2	0.0000	-0.1098	0.00
SddC	0.0000	0.5315	0.00
StN	0.0000	0.1020	0.00
SsNH2_acnt	0.0000	0.7574	0.00
SdsssP_acnt	0.0000	1.0683	0.00
SaaS_acnt	0.0000	-0.9442	0.00
Qsv	0.6321	0.6629	0.42
MDEN12	0.0000	-0.3705	0.00
BEHm1	4.6267	0.3299	1.53
TIE	268.6655	-0.0008	-0.23
ATS5p	4.0726	0.1089	0.44
MATS6m	-0.0105	-0.2993	0.00
MATS4v	-0.1053	-0.2083	0.02
MATS6v	0.0200	0.5849	0.01
MATS8v	-0.0542	0.4000	-0.02
GATS1p	1.4306	-0.3492	-0.50
GATS3p	0.7899	0.3034	0.24
SRW07	0.0000	0.0014	0.00
ALOGP	-2.2243	0.5107	-1.14
=C [aliphatic attach]	0.0000	0.3600	0.00
-O- [phosphorus attach]	0.0000	0.8688	0.00
-OH [aromatic attach]	0.0000	0.2668	0.00
-CH=N	0.0000	1.0449	0.00
-C(=O)O- [aliphatic attach]	0.0000	0.4000	0.00
-C(=O)O- [nitrogen attach]	0.0000	0.9850	0.00
-CCl3 [aliphatic attach]	0.0000	0.7305	0.00
Model intercept	1.0000	0.9039	0.9039
Predicted value -Log10(mol/L)			1.69

3.3. Predicted T. pyriformis IGC50 (48 hr)

Prediction results

Endpoint	Experimental value	Predicted value[b]	Prediction interval
T. pyriformis IGC_{50} (48 hr) -Log10(mol/L)	N/A	N/A	N/A
T. pyriformis IGC_{50} (48 hr) mg/L	N/A	N/A	N/A

[b]A prediction cannot be made

3.4. Predicted Oral rat LD50

Prediction results

Endpoint	Experimental value	Predicted value[b]	Prediction interval
Oral rat LD_{50} -Log10(mol/kg)	N/A	N/A	N/A
Oral rat LD_{50} mg/kg	N/A	N/A	N/A

[b]A prediction cannot be made

3.5. *Predicted Bioconcentration factor*

Prediction results

Endpoint	Experimental value	Predicted value
Bioconcentration factor Log10	N/A	0.39
Bioconcentration factor	N/A	2.44

Cluster model predictions and statistics

Cluster model	Test chemical descriptor values	Prediction interval Log10	r^2	q^2	#chemicals	Applicability Domain
1079	Descriptors	0.91 ± 1.25	0.748	0.715	343	OK
1080	Descriptors	-0.18 ± 1.33	0.764	0.733	540	OK

Model # 1079

Parameter	Value
Endpoint	Bioconcentration factor
r^2	0.748
q^2	0.715
Number of chemicals	343
Model	1079

Model fit results

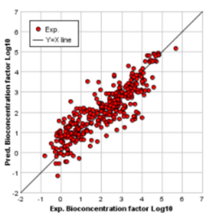

Model coefficients

Coefficient	Definition	Value	Uncertainty*
xvp10	Valence 10th order path chi index	-0.9711	0.3808
SdO_acnt	Count of (= O) (SdO_acnt)	-0.3265	0.1339
SHsNH2	Sum of ([? NH2]) hydrogen E-States (SHsNH2)	-0.1621	0.1023
icycmm	Mean information on the vertex cycle matrix magnitude	0.1450	0.0607
MDEC13	Molecular distance edge between all primary and tertiary carbons	0.0849	0.0301
MDEN22	Molecular distance edge between all secondary nitrogens	-0.2066	0.1099
BEHp2	Highest eigenvalue n. 2 of Burden matrix / weighted by atomic polarizabilities	1.4370	0.3704
MAXDN	Maximal electrotopological negative variation	0.1665	0.0529
nDB	Number of double bonds	-0.2134	0.0990
MATS1v	Moran autocorrelation - lag 1 / weighted by atomic van der Waals volumes	1.1491	0.2153
GATS5v	Geary autocorrelation - lag 5 / weighted by atomic van der Waals volumes	-0.3254	0.1647
GATS3p	Geary autocorrelation - lag 3 / weighted by atomic polarizabilities	0.5738	0.1874
Ui	unsaturation index	-0.3154	0.0875
-Cl [aromatic attach]	-Cl [aromatic attach] fragment count	0.3098	0.0567
-C(=O)- [2 aromatic attach]	-C(=O)- [2 aromatic attach] fragment count	0.4964	0.2100
-COOH [aliphatic attach]	-COOH [aliphatic attach] fragment count	-0.6537	0.3452
Model intercept	Intercept of multilinear regression model	-3.1893	1.1958

* value for 90% confidence interval

Model equation:
Bioconcentration factor = -0.9711×(xvp10) - 0.3265×(SdO_acnt) - 0.1621×(SHsNH2) + 0.1450×(icycmm) + 0.0849×(MDEC13) - 0.2066×(MDEN22) + 1.4370×(BEHp2) + 0.1665×(MAXDN) - 0.2134×(nDB) + 1.1491×(MATS1v) - 0.3254×(GATS5v) + 0.5738×(GATS3p) - 0.3154×(Ui) + 0.3098×(-Cl [aromatic attach]) + 0.4964×(-C(=O)- [2 aromatic attach]) - 0.6537×(-COOH [aliphatic attach]) - 3.1893

Descriptor Values

Descriptor	Value	Coefficient	Value × Coefficient
xvp10	0.0991	-0.9711	-0.10
SdO_acnt	3.0000	-0.3265	-0.98
SHsNH2	0.0000	-0.1621	0.00
icycmm	3.5850	0.1450	0.52
MDEC13	2.1260	0.0849	0.18
MDEN22	0.0000	-0.2066	0.00
BEHp2	3.7879	1.4370	5.44
MAXDN	6.3019	0.1665	1.05
nDB	3.0000	-0.2134	-0.64
MATS1v	-0.2058	1.1491	-0.24
GATS5v	0.9665	-0.3254	-0.31
GATS3p	0.7899	0.5738	0.45
Ui	2.0000	-0.3154	-0.63
-Cl [aromatic attach]	0.0000	0.3098	0.00
-C(=O)- [2 aromatic attach]	0.0000	0.4964	0.00
-COOH [aliphatic attach]	1.0000	-0.6537	-0.65
Model intercept	1.0000	-3.1893	-3.1893
Predicted value Log10			0.91

Model # 1080

Parameter	Value
Endpoint	Bioconcentration factor
r^2	0.764
q^2	0.733
Number of chemicals	540
Model	1080

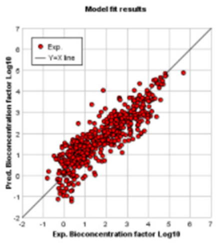

Model coefficients

Coefficient	Definition	Value	Uncertainty*
knotp	Difference between chi cluster-3 and chi path/cluster-4	0.1788	0.0708
xvp9	Valence 9th order path chi index	-0.8222	0.2579
SsssCH	Sum of (> CH ?) E-States (SsssCH)	0.1245	0.0923
SdO_acnt	Count of (= O) (SdO_acnt)	-0.2765	0.0699
Hmin	Minimum hydrogen E-State value in molecule.	0.4088	0.1451
SHHBd	Sum of E-State indices for hydrogen bond donors	-0.0593	0.0318
BEHm2	Highest eigenvalue n. 2 of Burden matrix / weighted by atomic masses	0.0727	0.0653
BEHp2	Highest eigenvalue n. 2 of Burden matrix / weighted by atomic polarizabilities	0.4236	0.2800
BEHp6	Highest eigenvalue n. 6 of Burden matrix / weighted by atomic polarizabilities	0.2024	0.1776
nN	Number of Nitrogen atoms	-0.1026	0.0507
MATS1v	Moran autocorrelation - lag 1 / weighted by atomic van der Waals volumes	0.5102	0.1273
MATS5v	Moran autocorrelation - lag 5 / weighted by atomic van der Waals volumes	0.2543	0.0958
MATS8e	Moran autocorrelation - lag 8 / weighted by atomic Sanderson electronegativities	-0.1279	0.1176
SRW10	Self-returning walk count of order 10	0.0001	0.0000
piID	Conventional bond order id number	-0.0084	0.0024
ALOGP	Ghose-Crippen octanol water coefficient	0.3447	0.0865
ALOGP2	Ghose-Crippen octanol water coefficient squared	-0.0197	0.0067
-Cl [aromatic attach]	-Cl [aromatic attach] fragment count	0.2072	0.0453
-COOH [aliphatic attach]	-COOH [aliphatic attach] fragment count	-0.4934	0.2361
-COOH [aromatic attach]	-COOH [aromatic attach] fragment count	-0.5709	0.2221
Model intercept	Intercept of multilinear regression model	-1.3347	0.6777

* value for 90% confidence interval

Model equation:
Bioconcentration factor = 0.1788×(knotp) - 0.8222×(xvp9) + 0.1245×(SsssCH) - 0.2765×(SdO_acnt) + 0.4088×(Hmin) - 0.0593×(SHHBd) + 0.0727×(BEHm2) + 0.4236×(BEHp2) + 0.2024×(BEHp6) - 0.1026×(nN) + 0.5102×(MATS1v) + 0.2543×(MATS5v) - 0.1279×(MATS8e) + 0.0001×(SRW10) - 0.0084×(piID) + 0.3447×(ALOGP) - 0.0197×(ALOGP2) + 0.2072×(-Cl [aromatic attach]) - 0.4934×(-COOH [aliphatic attach]) - 0.5709×(-COOH [aromatic attach]) - 1.3347

Descriptor Values

Descriptor	Value	Coefficient	Value × Coefficient
knotp	-13.4132	0.1788	-2.40
xvp9	0.2310	-0.8222	-0.19
SsssCH	0.0000	0.1245	0.00
SdO_acnt	3.0000	-0.2765	-0.83
Hmin	0.9689	0.4088	0.40
SHHBd	20.3675	-0.0593	-1.21
BEHm2	4.0255	0.0727	0.29
BEHp2	3.7879	0.4236	1.60
BEHp6	3.1622	0.2024	0.64
nN	0.0000	-0.1026	0.00
MATS1v	-0.2058	0.5102	-0.10
MATS5v	0.0260	0.2543	0.01
MATS8e	-0.0867	-0.1279	0.01
SRW10	90626.0000	0.0001	5.23
piID	112.1908	-0.0084	-0.94
ALOGP	-2.2243	0.3447	-0.77
ALOGP2	4.9475	-0.0197	-0.10
-Cl [aromatic attach]	0.0000	0.2072	0.00
-COOH [aliphatic attach]	1.0000	-0.4934	-0.49
-COOH [aromatic attach]	0.0000	-0.5709	0.00
Model intercept	1.0000	-1.3347	-1.3347
Predicted value Log10			-0.18

3.6. Developmental Toxicity

Prediction results

Endpoint	Experimental value	Predicted value[b]
Developmental Toxicity value	N/A	N/A
Developmental Toxicity result	N/A	N/A

[b]No prediction could be made

3.7. Predicted Mutagenicity

Prediction results

Endpoint	Experimental value	Predicted value
Mutagenicity value	N/A	0.32
Mutagenicity result	N/A	Mutagenicity Negative

Cluster model predictions and statistics

Cluster model	Test chemical descriptor values	Predicted value	Concordance	Sensitivity	Specificity	#chemicals	Applicability Domain
9156	Descriptors	0.35	0.916	0.763	1.000	107	OK
9160	Descriptors	0.43	0.899	0.737	0.986	109	OK
9184	Descriptors	0.19	0.908	0.804	0.958	142	OK

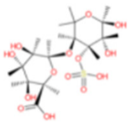

Cluster models with applicability domain violation

Cluster model	Test chemical descriptor values	Predicted value	Concordance	Sensitivity	Specificity	#chemicals	Applicability Domain
8954	Descriptors	0.57	1.000	1.000	1.000	10	Rmax constraint not met
8989	Descriptors	-0.60	1.000	1.000	1.000	16	Rmax constraint not met
9179	Descriptors	-1.32	0.920	0.786	0.979	137	Model ellipsoid constraint not met

Model # 9156

Parameter	Value
Endpoint	Mutagenicity
Concordance	0.916
Sensitivity	0.763
Specificity	1.000
Number of chemicals	107
Model	9156

Model coefficients

Coefficient	Definition	Value	Uncertainty*
SaaaC_acnt	Count of (aaaC) (SaaaC_acnt)	-0.1078	0.0330
numwHBd	Number of weak hydrogen bond donors (i.e. –CHX, where X = Cl, F)	0.1975	0.1206
BELm4	Lowest eigenvalue n. 4 of Burden matrix / weighted by atomic masses	-2.0214	0.6078
BELp3	Lowest eigenvalue n. 3 of Burden matrix / weighted by atomic polarizabilities	1.2901	0.9658
nR10	Number of 10-membered rings	0.0578	0.0425
GATS7v	Geary autocorrelation - lag 7 / weighted by atomic van der Waals volumes	-0.2141	0.1533
-CH< [aromatic attach]	-CH< [aromatic attach] fragment count	0.1091	0.1038
=C [aliphatic attach]	=C [aliphatic attach] fragment count	-0.0784	0.0653
-F [aliphatic attach]	-F [aliphatic attach] fragment count	-0.3145	0.2412
-O- [oxygen attach]	-O- [oxygen attach] fragment count	0.9290	0.3735
-O- [2 aromatic attach]	-O- [2 aromatic attach] fragment count	0.2546	0.1810
-NH- [nitrogen attach]	-NH- [nitrogen attach] fragment count	0.4193	0.2373
-C(=O)- [2 aromatic attach]	-C(=O)- [2 aromatic attach] fragment count	0.1072	0.0622
-C(=O)O- [cyclic]	-C(=O)O- [cyclic] fragment count	-0.3766	0.3117
Model intercept	Intercept of multilinear regression model	1.5339	1.4093

* value for 90% confidence interval

Model equation:
Mutagenicity = -0.1078×(SaaaC_acnt) + 0.1975×(numwHBd) - 2.0214×(BELm4) + 1.2901×(BELp3) + 0.0578×(nR10) - 0.2141×(GATS7v) + 0.1091×(-CH< [aromatic attach]) - 0.0784×(=C [aliphatic attach]) - 0.3145×(-F [aliphatic attach]) + 0.9290×(-O- [oxygen attach]) + 0.2546×(-O- [2 aromatic attach]) + 0.4193×(-NH- [nitrogen attach]) + 0.1072×(-C(=O)- [2 aromatic attach]) - 0.3766×(-C(=O)O- [cyclic]) + 1.5339

Descriptor Values

Descriptor	Value	Coefficient	Value × Coefficient
SaaaC_acnt	0.0000	-0.1078	0.00
numwHBd	0.0000	0.1975	0.00
BELm4	1.6503	-2.0214	-3.34
BELp3	1.8351	1.2901	2.37
nR10	0.0000	0.0578	0.00
GATS7v	1.0143	-0.2141	-0.22
-CH< [aromatic attach]	0.0000	0.1091	0.00
=C [aliphatic attach]	0.0000	-0.0784	0.00
-F [aliphatic attach]	0.0000	-0.3145	0.00
-O- [oxygen attach]	0.0000	0.9290	0.00
-O- [2 aromatic attach]	0.0000	0.2546	0.00
-NH- [nitrogen attach]	0.0000	0.4193	0.00
-C(=O)- [2 aromatic attach]	0.0000	0.1072	0.00
-C(=O)O- [cyclic]	0.0000	-0.3766	0.00
Model intercept	1.0000	1.5339	1.5339
Predicted value			0.35

Model # 9160

Parameter	Value
Endpoint	Mutagenicity
Concordance	0.899
Sensitivity	0.737
Specificity	0.986
Number of chemicals	109
Model	9160

Model coefficients

Coefficient	Definition	Value	Uncertainty*
SdssC_acnt	Count of (= C <) (SdssC_acnt)	-0.0338	0.0306
SaaaC_acnt	Count of (aaaC) (SaaaC_acnt)	-0.1628	0.0387
MDEC23	Molecular distance edge between all secondary and tertiary carbons	0.0169	0.0068
MDEN23	Molecular distance edge between all secondary and tertiary nitrogens	0.2630	0.2122
BELm4	Lowest eigenvalue n. 4 of Burden matrix / weighted by atomic masses	-1.0218	0.4695
BELp1	Lowest eigenvalue n. 1 of Burden matrix / weighted by atomic polarizabilities	3.3380	1.3808
GATS3p	Geary autocorrelation - lag 3 / weighted by atomic polarizabilities	0.4345	0.3241
-O- [oxygen attach]	-O- [oxygen attach] fragment count	0.7680	0.3864
-C(=O)- [2 aromatic attach]	-C(=O)- [2 aromatic attach] fragment count	0.2305	0.0635
Model intercept	Intercept of multilinear regression model	-5.5673	3.1301

* value for 90% confidence interval

Model equation:
Mutagenicity = -0.0338×(SdssC_acnt) - 0.1628×(SaaaC_acnt) + 0.0169×(MDEC23) + 0.2630×(MDEN23) - 1.0218×(BELm4) + 3.3380×(BELp1) + 0.4345×(GATS3p) + 0.7680×(-O- [oxygen attach]) + 0.2305×(-C(=O)- [2 aromatic attach]) - 5.5673

Descriptor Values

Descriptor	Value	Coefficient	Value × Coefficient
SdssC_acnt	1.0000	-0.0338	-0.03
SaaaC_acnt	0.0000	-0.1628	0.00
MDEC23	0.0000	0.0169	0.00
MDEN23	0.0000	0.2630	0.00
BELm4	1.6503	-1.0218	-1.69
BELp1	2.2084	3.3380	7.37
GATS3p	0.7899	0.4345	0.34
-O- [oxygen attach]	0.0000	0.7680	0.00
-C(=O)- [2 aromatic attach]	0.0000	0.2305	0.00
Model intercept	1.0000	-5.5673	-5.5673
Predicted value			0.43

Model # 9184

Parameter	Value
Endpoint	Mutagenicity
Concordance	0.908
Sensitivity	0.804
Specificity	0.958
Number of chemicals	142
Model	9184

Model coefficients

Coefficient	Definition	Value	Uncertainty*
SaaNH	Sum of (aaNH) E-States (SaaNH)	-0.0422	0.0307
SdssNp	Sum of (= N+ <) E-States (SdssNp)	-0.5950	0.3707
SaaaC_acnt	Count of (aaaC) (SaaaC_acnt)	-0.0449	0.0250
numwHBd	Number of weak hydrogen bond donors (i.e. –CHX, where X = Cl, F)	0.2750	0.1131
idm	Total information content on the distance magnitude	0.0000	0.0000
BELe5	Lowest eigenvalue n. 5 of Burden matrix / weighted by atomic Sanderson electronegativities	-0.6176	0.2662
MATS7p	Moran autocorrelation - lag 7 / weighted by atomic polarizabilities	0.2182	0.2110
-CH< [aromatic attach]	-CH< [aromatic attach] fragment count	0.2336	0.0734
=C [aliphatic attach]	=C [aliphatic attach] fragment count	-0.0718	0.0543
-O- [oxygen attach]	-O- [oxygen attach] fragment count	0.7999	0.3704
-C(=O)- [2 aromatic attach]	-C(=O)- [2 aromatic attach] fragment count	0.1814	0.0514
Model intercept	Intercept of multilinear regression model	1.0578	0.3755

* value for 90% confidence interval

Model equation:
Mutagenicity = -0.0422×(SaaNH) - 0.5950×(SdssNp) - 0.0449×(SaaaC_acnt) + 0.2750×(numwHBd) + 0.0000×(idm) - 0.6176×(BELe5) + 0.2182×(MATS7p) + 0.2336×(-CH< [aromatic attach]) - 0.0718×(=C [aliphatic attach]) + 0.7999×(-O- [oxygen attach]) + 0.1814×(-C(=O)- [2 aromatic attach]) + 1.0578

Descriptor Values

Descriptor	Value	Coefficient	Value × Coefficient
SaaNH	0.0000	-0.0422	0.00
SdssNp	0.0000	-0.5950	0.00
SaaaC_acnt	0.0000	-0.0449	0.00
numwHBd	0.0000	0.2750	0.00
idm	30874.8949	0.0000	0.01
BELe5	1.4061	-0.6176	-0.87
MATS7p	-0.0510	0.2182	-0.01
-CH< [aromatic attach]	0.0000	0.2336	0.00
=C [aliphatic attach]	0.0000	-0.0718	0.00
-O- [oxygen attach]	0.0000	0.7999	0.00
-C(=O)- [2 aromatic attach]	0.0000	0.1814	0.00
Model intercept	1.0000	1.0578	1.0578
Predicted value			0.19
